# TET3 protects the *Dlk1-Dio3* imprinted locus from DNA hypomethylation during adult NSC reprogramming

**DOI:** 10.1016/j.isci.2025.113994

**Published:** 2025-11-12

**Authors:** Laura Lázaro-Carot, Esteban Jiménez-Villalba, Jordi Planells, Anna Lozano-Ureña, Jennifer Díaz-Moncho, Raquel Montalbán-Loro, Adela Lleches-Padilla, Martina Kirstein, Mitsuteru Ito, Elizabeth J. Radford, Sacri R. Ferrón

**Affiliations:** 1 Instituto de Biotecnología y Biomedicina (BiotecMed)/Departamento de Biología Celular, Universidad de Valencia, 46100 Valencia, Spain; 2 Department of Genetics, University of Cambridge, Cambridge CB2 3EH, UK; 3 Department of Pediatrics, University of Cambridge, Cambridge CB2 0QQ, UK

**Keywords:** Epigenetics, Genomics, Molecular Genetics, Stem cells research, Transcriptomics

## Abstract

Genomic imprinting is an epigenetic mechanism that controls monoallelic expression according to parental origin. Imprinted genes are regulated by DNA methylation at imprinting control regions (ICRs), differentially methylated regions (DMRs) that distinguish between parental alleles. Cell reprogramming into induced pluripotent stem cells (iPSCs) offers a valuable model for studying pluripotency. Thus, discerning whether genomic imprinting changes during reprogramming represent epigenetic abnormalities or essential adaptations of pluripotency is crucial. Here, we integrate RNA-seq and MeDIP-seq analysis to profile mouse iPSCs derived from adult neural stem cells (NSCs). Our findings reveal that most ICRs undergo DNA hypomethylation in iPSCs, although the IG-DMR within the *Dlk1-Dio3* imprinted cluster remains methylated, serving as an epigenetic marker of pluripotency. We further identify a non-canonical role for the dioxygenase TET3 in maintaining IG-DMR methylation through the transcriptional regulation of *Oct4* and *Trim28*. These findings highlight genomic imprinting as a dynamic regulator of gene dosage during pluripotency acquisition.

## Highlights

• iPSCs derived from adult NSCs exhibit global loss of imprinting due to ICR hypomethylation
• The IG-DMR within the *Dlk1-Dio3* cluster resists hypomethylation during reprogramming
• TET3 dioxigenase maintains IG-DMR methylation through *Trim28* and *Oct4* transcriptional control
• Selective imprinting at the IG-DMR is essential for full pluripotency acquisition

## Graphical abstract

**Figure:**
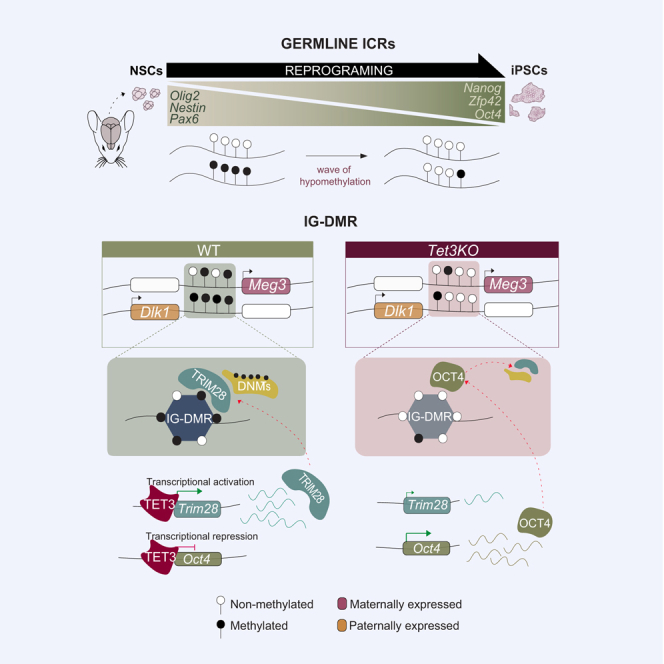

## Introduction

During mammalian development, the vast majority of genes are expressed or repressed from both alleles. However, there is a small number of genes, termed “*imprinted genes*” that are expressed monoallelicaly from either the maternally or the paternally inherited chromosomes.^1^ Approximately 150 imprinted genes have been described in mammals and are generally organized in clusters, although examples of singleton imprinted genes do exist.^2^ An imprinting cluster is usually under the control of a DNA element, called the imprinting control region (ICR), that consists of differentially DNA methylated regions (DMRs) between the two parental chromosomes.^3^^,^^4^ Deletion or alteration of an ICR results in loss of imprinting (LOI) of multiple genes in the cluster, and this has been associated with several human pathologies and imprinting disorders such as Prader-Willi Syndrome (PWS) and Angelman syndrome.^2^ The parental specific marks at ICRs are established in the developing germline, resulting as a result gametes bearing imprints according to the sex of the individual.^1^^,^^3^ After fertilization, a rapid and extensive reprogramming of the parentally inherited genomes occurs, and most DNA methylation is lost.^5^ However, the parental-specific imprints are maintained during this period, and a memory of parental origin is propagated into daughter cells during somatic cell divisions.^6^ At a molecular level, DNA methylation is a dynamic process where different enzymes are involved in actively methylating and demethylating cytosine residues of the DNA. Methylation is established and maintained by the DNA methyltransferase (DNMT) family of enzymes, either *de novo* by DNMT3A and DNMT3B, or maintained by DNMT1. On the other hand, the ten-eleven translocases (TET) enzyme family, integrated by TET1, TET2, and TET3, catalyzes the conversion of 5-methylcytosine (5mC) into 5-hydroxymethylcytosine (5hmC) to remove methylation marks.^7^ TET proteins have been implicated in maintaining DNA methylation at ICRs during the germline resetting of genomic imprints in embryonic development.^8^

Beyond their well-established catalytic function in DNA demethylation, TET proteins have been implicated in non-canonical roles that do not rely on their enzymatic activity. TET proteins can bind preferentially to 5mC-free promoters and interact with chromatin regulators such as histone deacetylases, acetyltransferases, Polycomb repressive complex 2, and O-linked N-acetylglucosamine transferase (*Ogt*) to modulate transcriptional programs.^9^ Additional evidence indicates that non-catalytic forms of TET proteins are functionally relevant, as they can rescue the proliferative phenotype of *Tet2-*deficient cells,^10^ and overexpression of either wild-type or catalytically inactive TET1 in the mouse hippocampus leads to the upregulation of memory-associated genes.^11^ In line with these findings, we have previously demonstrated that TET3 regulates the transcription of the imprinted gene *Snrpn* through a non-catalytic mechanism, further supporting the existence of non-conventional functions for TET proteins.^12^

*In vitro* reprogramming of somatic cells into induced pluripotent stem cells (iPSCs), has enormous therapeutic potential as it has opened up the possibility of generating patient-specific pluripotent cell lines to study and treat different degenerative diseases. A variety of cell types have been reprogrammed into iPSCs, including fibroblasts, hepatocytes, gastric epithelial cells, B cells, pancreatic β cells, neural progenitor cells, melanocytes, or keratinocytes.^13^^,^^14^^,^^15^^,^^16^^,^^17^ Different combinations of reprogramming factors, such as *Oct4 (Pou5f1)*, *Sox2*, *Klf4,* and *c-Myc (Myc)*, have been used to convert somatic cells into iPSCs with comparable expression profiles to embryonic stem cells (ESCs).^17^ The key determinants and the temporal sequence of epigenetic events that transition a differentiated cell to a pluripotent state during iPSC derivation remain incompletely understood.

Evidence from multiple studies highlights the critical role of DNA methylation dynamics in successful reprogramming, particularly the requirement for demethylation at promoters of pluripotency-associated genes.^14^ Incomplete DNA demethylation can result in only partially reprogrammed cells that retain somatic memory, as the erasure of differentiation-specific epigenetic marks is required for faithful reprogramming.^18^^,^^19^ Although genomic imprinting remains relatively stable in somatic cells, it is frequently disrupted during iPSC reprogramming, with certain imprinted regions being more susceptible to loss of imprinting than others.^20^^,^^21^^,^^22^^,^^23^^,^^24^ Consistently, aberrant silencing of imprinted genes during reprogramming has been linked to impaired developmental potential in iPSCs.^23^^,^^25^ A well-characterized example is the *Dlk1-Dio3* imprinted domain on murine chromosome 12, where the transcriptional repression of maternally expressed genes such as *Meg3*, *Rian*, and *Mirg* has been observed in many iPSC clones.^16^^,^^22^^,^^26^^,^^27^ This silencing correlates with reduced ability to generate high-grade chimeras or fully iPSC-derived mice.^26^ In parallel, *Grb10*, another imprinted gene, has been identified as highly expressed in fully reprogrammed nuclear transfer ESCs (ntESCs), suggesting a role in the establishment or maintenance of full pluripotency.^28^ More recently, *H19*, an imprinted long non-coding RNA, has been shown to modulate reprogramming efficiency as its knockdown enhances the generation of ESC-like iPSC colonies by increasing the expression of core pluripotency genes such as *Oct4*, *Nanog*, and *Rex1.*^29^ These findings suggest that specific imprinted loci may actively influence the epigenetic remodeling required to reach a fully reprogrammed pluripotent state. Notably, these imprinting defects in iPSCs appear to be influenced by factors such as the sex of the donor cells and *in vitro* culture conditions, and these alterations are not corrected upon differentiation.^20^^,^^30^ Therefore, distinguishing between essential and undesirable imprinting changes occurring during the acquisition of pluripotency remains crucial for improving the functional quality of iPSCs and ensuring their safety in therapeutic applications.

Here, we present a comprehensive and unbiased analysis of transcriptomic and epigenomic alterations in iPSCs generated from adult NSCs using only the reprogramming factors *Klf4* and *Oct4.* Genome-wide RNA-seq and MeDIP-seq revealed extensive transcriptional reprogramming accompanied by widespread DNA hypomethylation in iPSCs. Notably, the majority of DMRs within established ICRs displayed significant hypomethylation, consistent with a global loss of genomic imprinting in these cells. However, the IG-DMR regulating the *Dlk1-Dio3* imprinting cluster on chromosome 12 retained its methylation, suggesting a locus-specific protective mechanism. We propose a previously uncharacterized role for TET3 in preserving IG-DMR methylation during reprogramming. Mechanistically, TET3 binds to the *Trim28* promoter, enhancing its transcription, and to the *Oct4* promoter, repressing its expression. TRIM28 (also known as KAP1 or TIF1-β) is a transcriptional co-repressor that recruits epigenetic modifiers, including DNMTs, to maintain DNA methylation at ICRs. In the absence of TET3, decreased *Trim28* expression facilitates OCT4 binding to the IG-DMR, displacing TRIM28 and resulting in loss of methylation at this locus. These findings identify TET3 as a critical regulator of imprinting stability and uncover a transcriptional-epigenetic axis essential for safeguarding selective epigenetic marks during the acquisition of pluripotency.

## Results

### Neural stem cells from the adult subventricular zone convert into a pluripotent state only with the transduction of *Oct4* and *Klf4*

**Figure 1:**
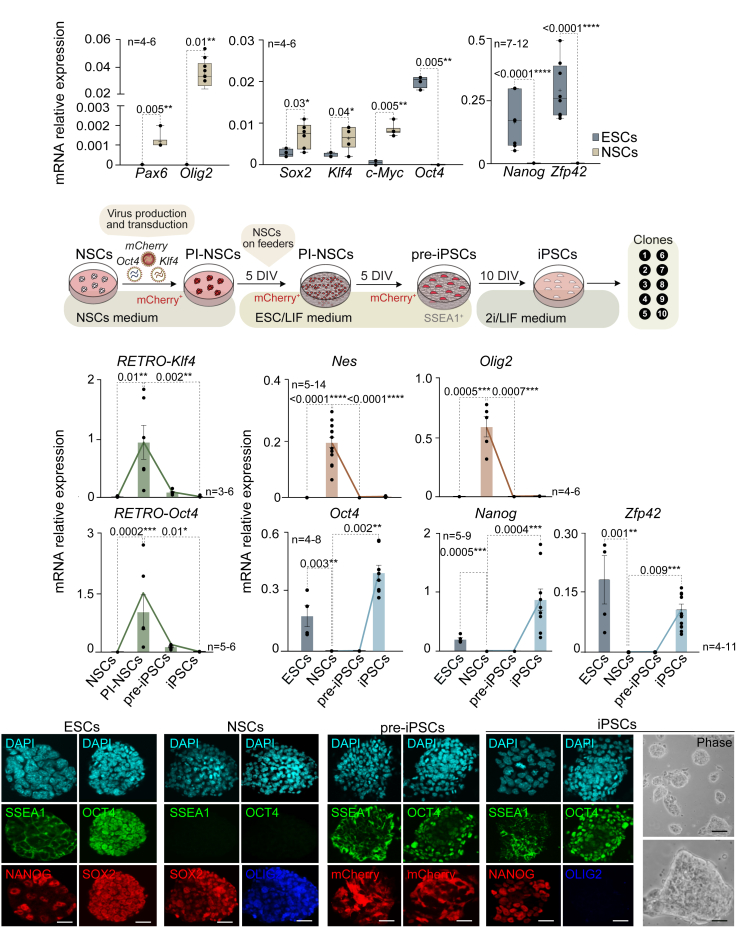
NSCs from the adult SVZ are reprogrammed into iPSCs by the exogenous expression of *Oct4* and *Klf4* (A) Quantitative PCR (qPCR) of the neural genes *Pax6* and *Olig2,* and the pluripotency genes *Nanog* and *Zfp42* in ESCs and adult NSCs. qPCR analysis of the endogenous expression of the reprogramming transcription factors *Oct4*, *Sox2*, *Klf4,* and *c-Myc* in ESCs and adult NSCs is also shown. (B) Schematic representation of the protocol used to reprogram adult NSCs into iPSCs. NSCs were infected with retroviruses encoding *Oct4*, *Klf4,* and the fluorescent protein mCherry. After five days *in vitro* (DIV) in NSCs medium, neurospheres formed by post-infected NSCs (PI-NSCs) were dissociated into single cells and plated on murine embryonic fibroblasts using ESC/LIF medium. Five days after dissociation, mCherry^+^ and SSEA1^+^ clone-like aggregates containing pre-iPSCs started to appear. Medium was then changed to 2i/LIF medium to complete the reprogramming process. After ten more DIVs, cells became full iPSCs, and ten single clones of each culture were picked and subcultured for further analysis. (C) qPCR analysis of retroviral *Klf4* and *Oct4* expression in adult NSCs, PI-NSCs, pre-iPSCs, and iPSCs. (D) qPCR analysis of the neural genes *Nes* and *Olig2* (upper panel) and the pluripotency-related genes *Oct4*, *Nanog, and Zfp42* (lower panel) in NSCs, pre-iPSCs, and iPSCs. ESCs were used as a control of the pluripotent state. (E) Immunocytochemistry (ICC) images of SSEA1, OCT4 (green) and SOX2 (red) in ESCs and adult NSCs. ICC for the pluripotency marker NANOG (red) in ESCs and for the neural marker OLIG2 in NSCs are also shown. (F) ICC images for SSEA1 and OCT4 (green) in pre-iPSCs and iPSCs. mCherry fluorescence in pre-iPSCs, ICC for the pluripotency marker NANOG (red) and the neural marker OLIG2 (blue) in iPSCs (middle panel) are also shown. Phase contrast images for fully reprogrammed iPSC clones are also included. *Gapdh* was used as a housekeeping gene for qPCR normalization. DAPI was used to counterstain nuclei in immunofluorescence images. Scale bars in E and F: 20 μm; phase contrast images in F: 40 μm (upper panel) and 5 μm (lower panel). Significance was evaluated using unpaired two-tailed *t* test, Mann-Whitney, ANOVA, and Kruskal-Wallis tests. *p*-values and number of samples are indicated. ∗*p* < 0.05; ∗∗*p* < 0.01; ∗∗∗*p* < 0.001; ∗∗∗∗*p* < 0.0001, n.s.: non significant. In box and whisker plots, the mean is marked with a (+) and whiskers represent the minimum and maximum values. In bar plots, mean and s.e.m. are shown. Each dot represents an independent culture. See also Figure S1.

Previous studies have reported that neurosphere cultures obtained from postnatal day 5 mouse brains endogenously express *Sox2*, *c-Myc* and *Klf4*. Thus, these cultures can be reprogrammed with *Oct4* alone, or with *Oct4* and *Klf4* at a similar efficiency to the reprogramming rate of murine fibroblast with the original four factors.^17^ To assess the expression level of these transcription factors in NSCs derived from the adult subventricular zone (SVZ), quantitative PCR (qPCR) was performed using a cell line of pluripotent embryonic stem cells (ESCs) as a reference. We found that adult NSCs consistently expressed neural genes such as *Pax6* or *Olig2*, and also expressed high levels of *Sox2*, *Klf4*, and *c-Myc* (Figure 1A). As expected, genes associated with pluripotency, such as *Oct4*, *Nanog,* or *Zfp42,* were not expressed in adult NSCs (Figure 1A). Based on this gene expression profile, we developed a reprogramming protocol using retroviral vectors encoding only for the transcription factors *Oct4* and *Klf4* (2 factors condition, 2F), along with a retrovirus encoding for the red fluorescent protein *mCherry* to track the exogenous expression of the reprogramming factors (Figure 1B). These retroviruses were produced by transfecting Plat-E packing cells with the retroviral plasmids (Figures 1B and S1A). Post-infected NSCs (PI-NSCs) were grown on a feeder layer of mouse embryonic fibroblasts using a medium supplemented with the cytokine leukemia inhibitory factor (LIF) (Figure 1B). After 10 days, cultures started to form clone-like aggregates, which were large, with poorly defined edges, and some of them expressed the pluripotency marker stage-specific embryonic antigen 1 (SSEA1) (Figures 1B and S1A). Retroviral vectors are transcriptionally silent in pluripotent stem cells; however, most of the clones formed were still positive for mCherry, indicating that despite expressing SSEA1, they were only partially reprogrammed (Figure S1A). Therefore, we considered this state of cells as pre-iPSCs (Figures 1B and S1A).^31^ To promote a ground state of pluripotency of these pre-iPSCs, we applied molecularly defined conditions by neutralizing inductive differentiation stimuli with a dual inhibition (2i) of mitogen-activated protein kinase signaling (MAPK) and glycogen synthase kinase-3 (GSK3).^32^ In this serum-free culture medium, LIF was also added to maximize clonogenic self-renewal of pluripotent cells (Figure 1B). This reprogramming protocol was repeated in six independent cultures of adult NSCs.^33^ After ten days in the new controlled 2i/LIF conditions, ESC-like colonies that did not express mCherry were obtained (Figure S1B), suggesting that cells had been fully reprogrammed into iPSCs. At least ten clones of each culture were isolated and expanded *in vitro* (Figure 1B).

To further characterize the acquisition of a pluripotent state of these clones, we performed a qPCR analysis on the PI-NSCs, pre-iPSCs, and the iPSCs generated. As expected, PI-NSCs expressed high levels of both *Oct4* and *Klf4* retroviral transgenes (Figure 1C). Although lower, the expression of retroviral genes was still present in pre-iPSCs (Figure 1C). Despite the undetectable expression of pluripotency markers such *as Oct4*, *Nanog,* and *Zfp42* in pre-iPSCs, the expression of neural-specific genes such as *Nes (Nestin)* and *Olig2* was completely downregulated in these cells (Figure 1D). These results confirmed that pre-iPSCs were an intermediate state in which critical attributes of true pluripotency, including the stable expression of endogenous *Oct4* and *Nanog,* had not been attained yet. Complete downregulation of retroviral transgenes, essential for full reprogramming, was corroborated in finally derived iPSCs compared to the original infected NSCs and to pre-iPSCs (Figure 1C). The expression of *Nes* and *Olig2* was completely absent in iPSCs (Figure 1D), and this was accompanied by the stable induction of endogenous pluripotency-related genes *Oct4, Nanog,* and *Zfp42* (Figure 1D), consistent with the acquisition of a pluripotent state. The presence of mCherry in pre-iPSCs but not in iPSCs was also confirmed (Figures S1A and S1B). Immunocytochemistry for OCT4 and NANOG, together with cytochemistry against alkaline phosphatase activity, confirmed the pluripotency of the generated iPSCs (Figures 1F and S1C).

We next corroborated the naive pluripotency of the iPSCs generated by evaluating the reactivation of the inactive X chromosome in female cultures.^34^ RNA levels of *Xist*, responsible for X chromosome inactivation, were significantly reduced in iPSCs, together with an increase in *Tsix* expression (Figure S1D), confirming the X chromosome reactivation and thus the acquisition of a fully pluripotent state. Moreover, genetic variations, such as aneuploidy or polyploidy, may be introduced during the generation of iPSCs.^35^ Karyotype analysis in different clones revealed that the majority of the analyzed lines (93%) exhibited a normal karyotype with around 40 chromosomes per metaphase (Figure S1E), and iPSC lines with chromosomal abnormalities were excluded from further studies.

### Induced pluripotent stem cells derived from adult neural stem cells differentiate *in vitro* and *in vivo* into cells from the three germ layers

**Figure 2:**
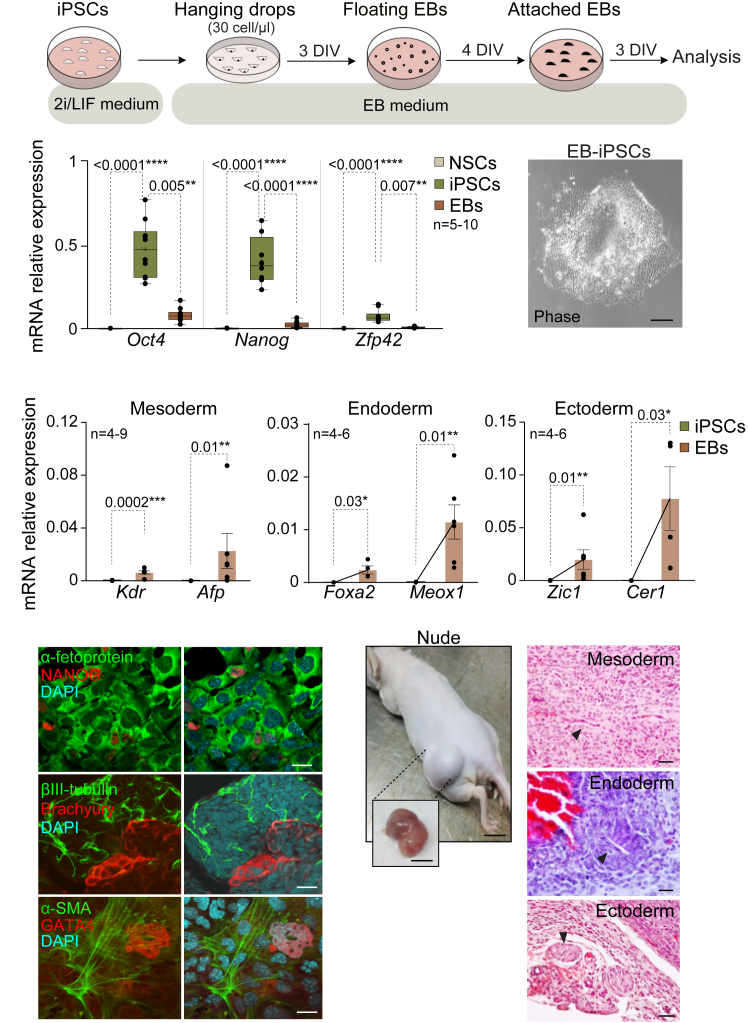
iPSCs generated from NSCs are able to differentiate into cells of the three germ layers *in vitro* and *in vivo* (A) Schematic representation of the embryoid bodies (EB) assay using the “*hanging drops*” method. iPSCs were dissociated, and the cell suspension (30 cells/μL) was distributed in drops in a plate that was incubated upside-down for three days *in vitro* (DIVs) in EB medium. Incipient EBs were incubated for four more DIVs in floating conditions. Then, EBs were seeded in gelatin pre-treated plates to allow differentiation for three more DIVs before analysis. (B) qPCR expression analysis of the pluripotency-related genes *Oct4*, *Nanog,* and *Zfp42* in NSCs, iPSCs, and iPSCs-derived EBs. Phase contrast image of a representative EB is shown. (C) qPCR analysis of *Kdr* and *Afp* (mesoderm), *Foxa2* and *Meox1* (endoderm), and *Zic1* and *Cer1* (ectoderm) in iPSCs and iPSCs-derived EBs. (D) ICC detection of the pluripotency marker NANOG (red) and the different germ layer markers: α-fetoprotein (green, endoderm), βIII-tubulin (green, ectoderm), and Brachyury (red, mesoderm); α-SMA (green, mesoderm) and GATA4 (red, endoderm) in iPSCs-derived EBs. (E) Image of the dorsolateral area of immunocompromised *Nude* mice two weeks after the injection of iPSCs, including a detailed image of the formed teratoma after its extraction (left panel). Histological analysis of teratomas using hematoxylin-eosin staining (right panel). Muscle fibers derived from mesoderm, columnar epithelium derived from endoderm, and epithelial cells derived from ectoderm are indicated with arrowheads. *Gapdh* was used as a housekeeping gene for qPCR normalization. DAPI was used to counterstain nuclei in immunofluorescence images. Scale bars in B: 10 μm; in D: 50 μm; and in E: 1 cm (left panel) and 20 μm (right panel). Significance was evaluated using Mann-Whitney, ANOVA, or Kruskal-Wallis tests. *P*-values and number of samples are indicated. ∗*p* < 0.05; ∗∗*p* < 0.01; ∗∗∗*p* < 0.001; ∗∗∗∗*p* < 0.0001, n.s.: non significant. In box and whisker plots, the mean is marked with a (+), and whiskers represent the maximum and minimum values. In bar plots, mean and s.e.m. are shown. Each dot represents an independent sample. See also Figure S2.

Pluripotent stem cells have the potential to differentiate into cells of the three germ layers: mesoderm, endoderm, and ectoderm. This differentiation potential is typically confirmed by demonstrating the capacity of the iPSCs to form three-dimensional structures called embryoid bodies (EBs), comprising characteristic cell types of these germ layers. EBs emulate the structure of developing embryos and serve as a model to obtain various cell lineages.^36^ Thus, to explore the developmental capacity of the iPSCs generated from adult NSCs, we induced EBs formation by subjecting cells to conditions that were adverse to pluripotency and proliferation, using the hanging drop method (Figure 2A).^36^ Suspended iPSCs on the dish lid aggregated at the base of the drop consistently generating uniform EBs (Figure 2A). ESCs and ESCs-derived EBs were used as a comparative condition of differentiation (Figure S2).

Initially, the gene expression levels of pluripotency and differentiation genes in iPSCs-derived EBs were determined using qPCR analysis. The results showed a downregulation of the pluripotency markers *Oct4*, *Nanog,* and *Zfp42* upon differentiation (Figure 2B). Additionally, a significant increase for the mesoderm markers *Kdr* and *Afp*, the endoderm markers *Foxa2* and *Meox1,* and the ectoderm markers *Zic1* and *Cer1* was observed, confirming the presence of cells from the three germ layers within the generated EBs (Figures 2C and S2A). The presence of differentiated cells was further confirmed by immunocytochemistry with specific antibodies against α-fetoprotein and GATA4 (endoderm), βIII-tubulin (neuroectoderm), and Brachyury and α-SMA (mesoderm) (Figures 2D and S2B). The *in vivo* pluripotent capacity of iPSCs derived from adult NSCs was next evaluated using the teratoma formation assay.^37^ Teratomas are non-malignant tumors that result from uncontrolled expansion and disorganized differentiation of pluripotent cells.^37^ In this study, adult NSCs-derived iPSCs were injected into the dorsolateral area in the subcutaneous space of *Nude* mice (Figure 2E). Subsequent histological analysis of the teratomas revealed disorganized tumoral cytoarchitecture and confirmed the presence of cells representing all three germ layers, such as ectodermal secretory epithelium, mesodermal cartilage, and endodermal gut epithelium derivatives (Figure 2E). Together, these findings confirmed the *in vitro* and *in vivo* pluripotency of the iPSCs derived from adult NSCs.

### Expression of imprinted genes is altered during the reprogramming of neural stem cells into induced pluripotent stem cells

**Figure 3:**
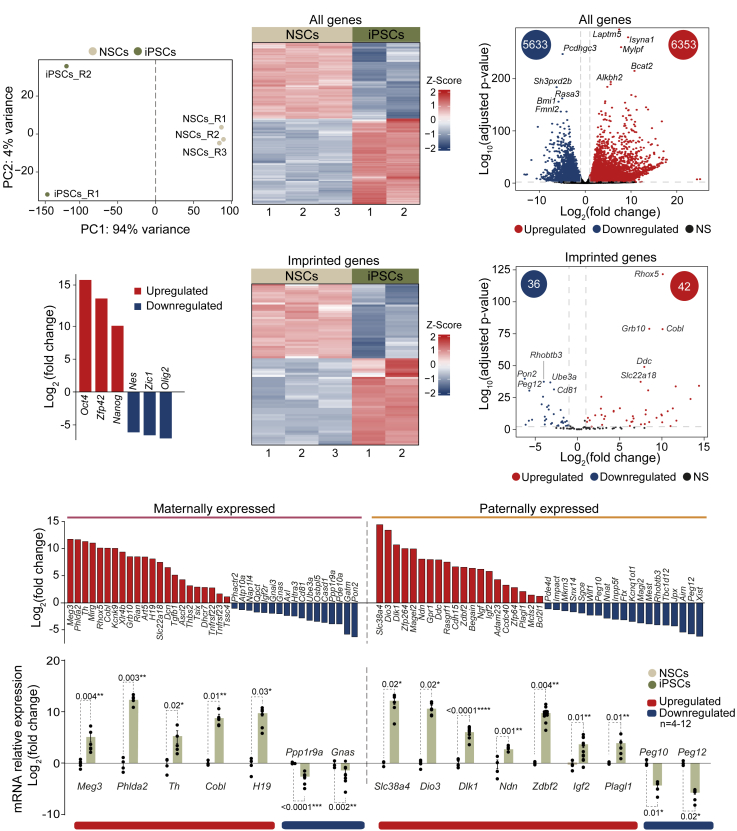
Expression of imprinted genes in adult NSCs is regulated during the reprogramming process (A) Principal component analysis (PCA) generated with the top 500 most variable genes obtained from RNA-seq of 3 NSCs and 2 iPSCs cultures, using normalized (variance stabilized transformation) gene counts. Principal components 1 and 2 are shown. (B) Heatmap displays the Z-score-scaled expression levels of all differentially expressed genes (DEGs) identified by comparing iPSCs and adult NSCs transcriptomes. (C) Volcano plot for all expressed genes based on RNA-seq data. The number of significantly downregulated (blue) and upregulated (red) genes in iPSCs relative to NSCs is indicated within the corresponding circles. (D) RNA-seq log_2_(fold change) in gene expression levels in iPSCs relative to NSCs for three pluripotency-associated genes, *Oct4, Zfp42,* and *Nanog,* and three neural lineage markers, *Nes*, *Zic1,* and *Olig2*. (E) Heatmap displays the Z-score-scaled expression levels of the imprinted DEGs in iPSCs relative to NSCs. (F) Volcano plot of all expressed imprinted genes based on RNA-seq data. The number of significantly downregulated (blue) and upregulated (red) genes in iPSCs relative to NSCs is indicated within the corresponding circles. (G) Representation of the log_2_(fold change) of all differentially expressed imprinted genes in iPSCs relative to NSCs. Maternally and paternally expressed genes are shown separately. (H) qPCR validation of selected imprinted DEGs identified by RNA-seq data. *Gapdh* was used to normalize gene expression data. Significance was evaluated using the unpaired two-tailed *t* test and the Mann-Whitney test. *P*-values and number of samples are indicated. ∗*p* < 0.05; ∗∗*p* < 0.01; ∗∗∗*p* < 0.001; ∗∗∗∗*p* < 0.0001, n.s.: non significant. In bar plots, mean and s.e.m. are shown. Each dot represents an independent culture. See also Figure S3.

To investigate the transcriptional changes associated with the reprogramming process, the transcriptome of both adult NSCs and derived iPSCs was profiled with RNA-seq (GSE282749). The principal component analysis (PCA) clearly distinguished iPSCs from NSCs (Figure 3A). Comparison of NSCs versus iPSCs unveiled 11,986 differentially expressed genes, representing around 37% of all genes expressed in NSCs (Figures 3B, 3C, and S3A). Among these alterations, 5,633 genes were downregulated (17,4% of all genes expressed in NSCs) while 6,353 were upregulated (19,6%) in iPSCs compared to adult NSCs (Figures 3C and S3A). Gene Ontology (GO) terms analysis showed several biological processes altered in iPSCs compared to NSCs (Figure S3B). Among upregulated genes, processes such as nucleotide metabolism, mitochondrial gene expression and translation, or biogenesis of ribonucleoprotein complexes were enriched; while downregulated genes showed enrichment in categories such as forebrain development, neurogenesis, and signal transduction (mainly Wnt signaling) (Figure S3B). As expected, RNA-seq analysis confirmed the upregulation of key pluripotency-associated genes in iPSCs compared to NSCs, including *Oct4, Zfp42,* and *Nanog* (Figure 3D). Conversely, neural-specific genes such as *Nes, Zic1, and Olig2* were downregulated (Figure 3D).

Some alterations of genomic imprinting have been observed during the reprogramming of somatic cells.^20^^,^^24^ These changes have an impact on stem cell plasticity, suggesting that genomic imprinting may be a mechanism employed to modulate gene dosage to control stem cell potential.^38^^,^^39^^,^^40^ Therefore, we next focused on the study of the regulation of imprinted genes during the reprogramming process using the RNA-seq data obtained in adult NSCs and iPSCs. We identified 78 imprinted genes that were differentially expressed between iPSCs and NSCs, representing 60% of all analyzed imprinted genes (Figures 3E, 3F, and S3A). Among them, similar changes in both paternally and maternally expressed genes were observed (Figure 3G). Imprinted gene expression changes were validated by qPCR in NSCs and iPSCs (Figure 3H), confirming that the acquisition of a pluripotent state also associates with significant transcriptional changes of several imprinted genes.

### Acquisition of pluripotency in adult neural stem cells requires global DNA hypomethylation

**Figure 4:**
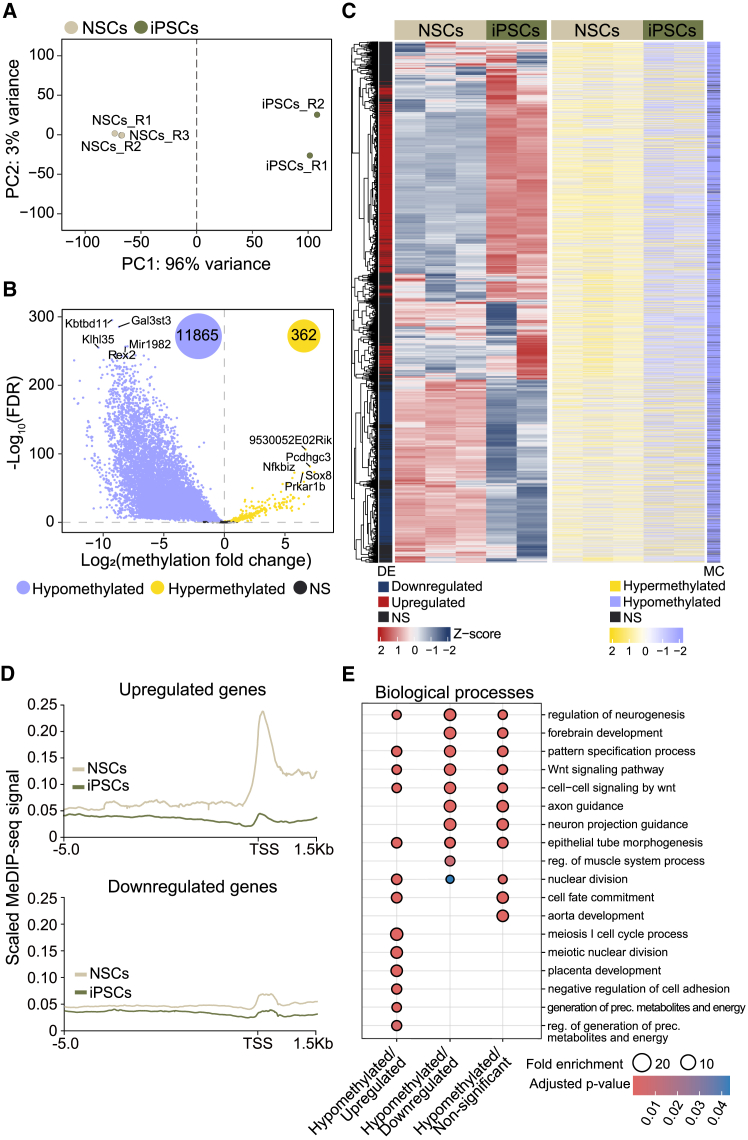
A global hypomethylation is observed in iPSCs' genome compared to adult NSCs (A) PCA from MeDIP-seq of three NSC and two iPSC cultures based on normalized (variance stabilized transformation) counts. (B) Volcano plot shows the differential methylation signal between iPSCs and NSCs. The number of significantly hypomethylated (purple) and hypermethylated (yellow) regions in iPSCs relative to NSCs is displayed. (C) Heatmap represents both normalized expression and methylation of the whole genome in NSCs and iPSCs. Differential expression (DE) is shown in red (upregulation) and blue (downregulation). Methylation changes (MCs) are shown in yellow (hypermethylation) and purple (hypomethylation). (D) Distribution of 5mC methylation signal around transcription start sites (TSS) of upregulated genes (upper panel) and downregulated genes (lower panel). (E) Gene ontology (GO) analysis of biological processes for gene groups identified with RNA-seq and MeDIP-seq data intersection. Dot size denotes the fold enrichment of each ontology over the background. DMR: differentially methylated region. Non-significant (NS) changes are shown in black. See also Figure S4.

DNA methylation represents one of the several epigenetic mechanisms employed by cells to regulate gene expression during cell fate decisions.^41^ Moreover, previous studies have reported that iPSCs exhibit lower levels of DNA methylation than somatic cells, highlighting that DNA demethylation might be a crucial chromatin feature for achieving pluripotency.^14^^,^^42^ In order to characterize methylation changes at a global level in adult NSCs and NSC-derived iPSCs, immunoprecipitation of methylated DNA using an antibody against 5-methylcytosine (5mC) followed by high-throughput sequencing (MeDIP-seq) was performed (GEO: GSE282748) (Figure S4A). The PCA of the results showed clear segregation between NSCs and iPSCs (Figure 4A). Consistent with this, MeDIP-seq analysis revealed genome-wide hypomethylation during the acquisition of the pluripotent state (Figures 4B, 4C, and S4B). Among the differentially methylated regions, 97% (11,865 regions) showed hypomethylation, whereas only 3% (362 regions) exhibited hypermethylation in iPSCs relative to NSCs (Figure 4B).

To explore the relationship between transcriptional changes and DNA methylation during reprogramming, RNA-seq and MeDIP-seq data were integrated (Figure 4C). The global hypomethylation found in iPSCs was particularly pronounced at transcription start sites (TSSs) and promoters, as demonstrated by overlapping methylation changes with different chromatin states (Figure S4C).^43^ Analysis of the methylation levels at promoter-proximal regions of differentially expressed genes showed that upregulated genes in iPSCs exhibited a more pronounced loss of methylation compared to downregulated genes, suggesting an inverse correlation between DNA methylation and gene expression (Figures 4D and S4D). Gene ontology (GO) analysis of the intersected data revealed that upregulated genes with hypomethylated promoters were enriched in biological processes related to cell division and metabolism, while downregulated genes with hypomethylated promoters were associated with neural development (Figure 4E). Importantly, a similar hypomethylation pattern was observed in ESCs relative to NSCs (Figures S4E and S4F), reflecting that global DNA hypomethylation represents a shared epigenetic feature of pluripotent states.

### IG-DMR escapes global DNA hypomethylation during the acquisition of pluripotency in adult neural stem cells

**Figure 5:**
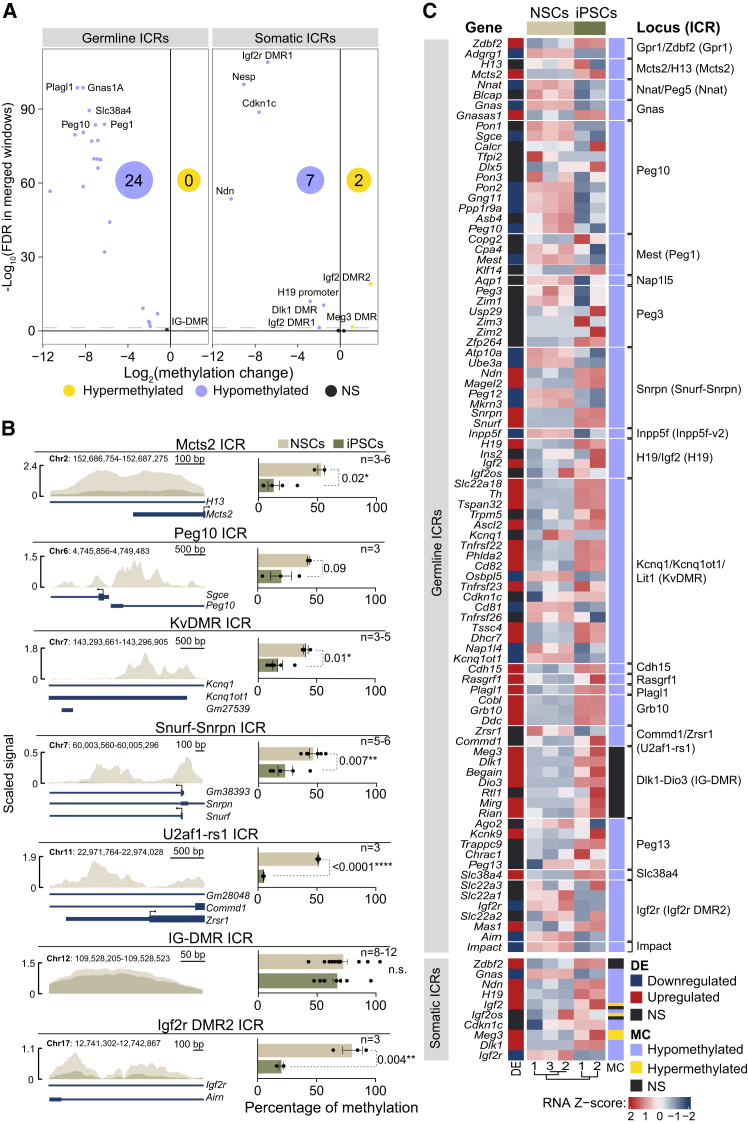
Alterations of methylation and gene expression are observed in different imprinting clusters in iPSCs (A) Volcano plot represents the methylation change between iPSCs and NSCs of different imprinting control regions (ICRs). The number of hypomethylated (purple) and hypermethylated (yellow) DMRs is shown. (B) MeDIP-seq methylation signal representation at ICR loci in NSCs and iPSCs (left panels). DNA methylation quantification by the pyrosequencing of bisulfite-converted DNA at specific ICRs in NSCs and iPSCs is shown (right panels). Genomic positions based on the Genome Reference Consortium Mouse Build 38 (GRCm38/mm10) are also indicated. (C) Heatmap represents both normalized expression (DE) and methylation (IMC) changes of imprinted clusters. Imprinted genes are cluster-organized, showing the methylation status of each ICR in iPSCs compared to NSCs. Significance was evaluated using the unpaired two-tailed *t* test and the Mann-Whitney test. *P*-values and number of samples are indicated. ∗*p* < 0.05; ∗∗*p* < 0.01; ∗∗∗*p* < 0.001; ∗∗∗∗*p* < 0.0001, n.s.: non significant. In bar plots, mean and s.e.m. are shown. Each dot represents an independent culture. See also Figure S5.

Imprinted loci are characterized by parent-of-origin-specific DNA methylation at ICRs, which is essential for maintaining monoallelic expression. These regions are particularly vulnerable to epigenetic alterations during cellular reprogramming, and dysregulation of imprinting has been implicated in developmental abnormalities and compromised pluripotency.^20^^,^^23^^,^^27^ Therefore, and given the variability in the extent and nature of the methylation changes at ICRs reported during pluripotency acquisition, we next analyzed the methylation dynamics across all described imprinted clusters during adult NSCs reprogramming. Consistent with the genome-wide patterns, both germline and somatic ICRs exhibited widespread hypomethylation in iPSCs compared to adult NSCs (Figure 5A).^44^ Specifically, MeDIP-seq analysis revealed that 24 out of the 25 germline ICRs (96% of all described gDMRs) and 7 out of the 13 somatic ICRs (54% of all described sDMRs) were hypomethylated in iPSCs (Figure 5A; Tables S1 and S2). However, IG-DMR, previously reported to be hypermethylated in adult NSCs, retained its hypermethylated status in iPSCs (Figures 5A and S5A).^38^ To validate these findings, bisulfite DNA treatment followed by pyrosequencing was performed in NSCs and iPSCs, corroborating the hypomethylation of most ICRs and the associated loss of imprinting in these clusters (Figure 5B). Importantly, the hypermethylation of the IG-DMR in both NSCs and iPSCs was also confirmed (Figures 5B, S5B, and S5C), indicating that this region resists the reprogramming-associated wave of DNA demethylation. Consistently, high levels of methylation at the IG-DMR were observed in ESCs (Figures S5B and S5C), indicating that this ICR remains epigenetically intact in the pluripotent state.

To investigate whether the observed changes in DNA methylation at ICRs were associated with alterations in imprinted gene expression, we compared transcriptomic and MeDIP-seq data in iPSCs and NSCs (Figure 5C). We found that 25% of the changes in imprinted gene expression correlated with the loss of methylation at ICRs in iPSCs (Figure 5C). Focusing on the IG-DMR, which regulates several genes within the *Dlk1-Dio3* cluster (Figure S5A), we observed that the expression levels of *Rtl1* remained unchanged in iPSCs compared to adult NSCs (Figure 5C).^38^^,^^45^ This is consistent with the fact that both cell types exhibit a hypermethylated IG-DMR, and this methylation state remains stable during reprogramming. In contrast, other genes within the cluster, including *Begain*, *Dlk1, Meg3,* and *Dio3,* were upregulated in iPSCs (Figure 5C). This locus also contains two somatic ICRs that regulate the imprinting of the paternally expressed gene *Dlk1* and the maternally expressed gene *Meg3* (Figures S5A and 5C). Both sDMRs are canonically methylated on the paternal allele; therefore, hypomethylation at the Dlk1 sDMR would not be expected to drive the upregulation of *Dlk1* observed in iPSCs. Additionally, the Meg3 sDMR was found to be hypermethylated in iPSCs, suggesting that the increased expression of *Meg3* is not directly linked to changes in methylation at its sDMR (Figures S5A and 5C). Interestingly, while the promoter region of *Meg3* and *Rtl1* showed no detectable methylation signal (Figure S5D), a significant hypomethylation was observed at the promoters of the upregulated genes *Begain*, *Dlk1,* and *Dio3* (Figure S5D). These findings suggest the existence of an additional transcriptional mechanism regulating gene expression within this cluster. Together, our results demonstrate that although the IG-DMR resists the widespread hypomethylation associated with reprogramming, the altered expression of genes within the *Dlk1-Dio3* locus likely arises from transcriptional mechanisms independent of changes in genomic imprinting.

To better assess the functional impact of IG-DMR methylation on pluripotency, we employed a global DNA demethylation approach using 5-azacytidine (5-AZA), a drug that induces widespread DNA demethylation acting through the irreversible inhibition of DNMTs (Figure S5E).^46^ 5-AZA-treated iPSCs showed significant hypomethylation at the IG-DMR compared to untreated iPSCs (Figure S5F). Importantly, cells exposed to 5-AZA also exhibited marked changes in gene expression. Specifically, we observed a downregulation of the core pluripotency marker *Nanog*, alongside an upregulation of *Nes*, a marker of neural lineage commitment (Figure S5G), confirming that hypomethylation at the IG-DMR compromises the pluripotent state of iPSCs and promotes a shift toward differentiation.

### TET3-mediated transcriptional regulation of *Trim28* preserves IG-DMR hypermethylation in induced pluripotent stem cells

**Figure 6:**
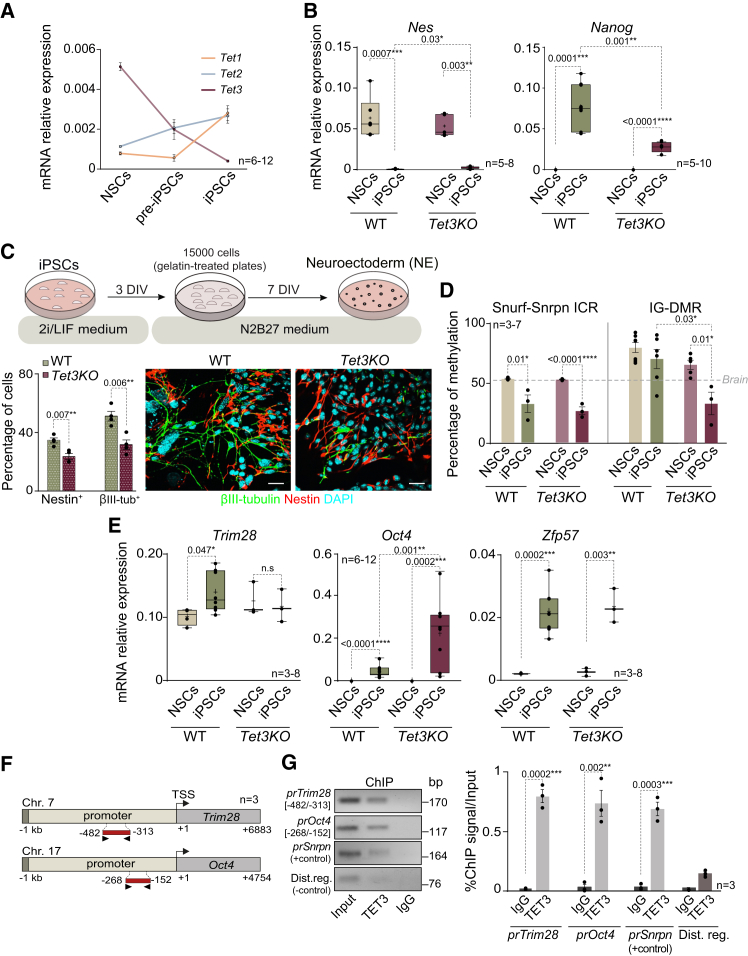
TET3 mediates IG-DMR methylation protection by regulating *Trim28* and *Oct4* gene expression (A) qPCR quantification of *Tet1*, *Tet2,* and *Tet3* in NSCs, pre-iPSCs, and iPSCs. (B) qPCR quantification of the neural marker *Nes* and the pluripotency marker *Nanog* in wild-type (WT) and *Tet3*-deficient (Tet3KO) NSCs and iPSCs. (C) Schematic of the protocol used to differentiate iPSCs into neuroectoderm (NE). iPSCs are disaggregated and re-plated in gelatin-treated plates at a 1.5 × 10^4^ cells/cm^2^ density in N2B27 supplemented medium. Seven days after cells are analyzed (upper panel). Percentage of Nestin and βIII-tubulin positive cells in iPSCs and NE cultures of both genotypes (lower left panel). Immunocytochemistry images of Nestin (red) and βIII-tubulin (green) in NE cultures of both genotypes (lower right panel). (D) Quantification by the pyrosequencing of the percentage of methylation at the Snurf-Snrpn ICR and at the IG-DMR in NSCs and iPSCs from both WT and Tet3KO cultures. Gray dashed line indicates the percentage of methylation in control brain samples. (E) qPCR analysis of *Trim28*, *Oct4* and *Zfp57* in NSCs and iPSCs from WT and Tet3KO mice. (F) Schematic representation of *Trim28* and *Oct4* promoter regions and amplicons used in the TET3 ChIP-qPCR assay. (G) qPCR analysis after ChIP with TET3 antibody in wild-type adult NSCs. *Trim28* and *Oct4* promoters were analyzed. The *Snrpn* promoter was used as a positive control for TET3 binding, while a distal, unrelated genomic region served as a negative control. Enrichment values represent the proportion of immunoprecipitated DNA relative to the input (%ChIP signal/input). *Gapdh* was used as a housekeeping gene for qPCR analysis. DAPI was used to counterstain DNA. Scale bars in C: 20 μm. Significance was evaluated using the unpaired two-tailed *t* test and the Mann-Whitney test. *P*-values and number of samples are indicated. ∗*p* < 0.05; ∗∗*p* < 0.01; ∗∗∗*p* < 0.001; ∗∗∗∗*p* < 0.0001, n.s.: non significant. In box and whisker plots, the mean is indicated as +, and whiskers represent the maximum and minimum values. In bar plots, mean and s.e.m. are shown. Each dot represents an independent culture. See also Figures S6–S9.

TET enzymes are dioxygenases that convert 5mC to 5hmC, resulting in the removal of methylation marks, and their role has been demonstrated to be critical for iPSCs reprogramming.^7^^,^^47^ Nevertheless, the specific functions of each TET enzyme in the context of reprogramming are still unclear. qPCR analysis showed that *Tet3* is the most abundant member of the *Tet* dioxygenases in NSCs isolated from the adult SVZ, and its levels are downregulated during the reprogramming process (Figures 6A and S6A).^12^ To investigate the potential role of TET3 in the demethylation process of ICRs during the acquisition of a pluripotent state, a murine genetic model with a conditional deletion of *Tet3* in *Gfap*-expressing cells (mainly NSCs and astrocytes) was generated. *Tet3* expression ablation was confirmed in NSCs isolated from the SVZ of *Tet3-*deficient (*Gfap-Tet3*^*KO*^) compared to wild-type (*Gfap-Tet3*^*WT*^) (Figure S6B). To induce reprogramming, *Gfap-Tet3*^*KO*^ and *Gfap-Tet3*^*WT*^ derived NSCs were co-transduced with the combination of *Oct4, Klf4,* and *mCherry*-encoding retroviral supernatants as previously described (Figure 1B). Pluripotency markers and silencing of neural genes were analyzed by qPCR in several iPSC clones, confirming the full reprogramming of *Tet3KO* NSCs into iPSCs (Figures 6B and S6C–S6E). Although the expression levels of the pluripotency-related gene *Zfp42* and the neural gene *Olig2* were indistinguishable between wild-type and *Tet3*-deficient iPSCs (Figure S6C), an incomplete upregulation of *Nanog* and downregulation of the neural marker *Nes* were observed in *Tet3*-deficient iPSCs compared to wild-type (Figure 6B). These results suggest that the absence of TET3 impairs the acquisition of full pluripotency of adult NSCs.

To determine whether *Tet3* deficiency affects the pluripotency of iPSCs, we induced EB formation using the hanging drop method (Figure 2A). Immunocytochemistry for Brachyury (T, mesoderm), α-fetoprotein (endoderm), and βIII-tubulin (neuroectoderm) on EBs confirmed that *Tet3KO* iPSCs could give rise to cells from all three germ layers (Figure S6F). Consistently, expression analysis of differentiation genes in *Tet3KO* EBs revealed a significant upregulation of mesoderm (*Kdr)*, endoderm (*Foxa2),* and ectoderm (*Cer1)* markers (Figure S6G). To further evaluate the differentiation potential of *Tet3-*deficient iPSCs, we directed their differentiation into neuroectoderm (NE) using LIF-free medium with N2 and B27 serum-free supplements (Figure 6C). qPCR analysis of the neural markers *Nes* and *Tubb3* showed that *Tet3KO* iPSCs failed to achieve physiological expression levels of these genes (Figure S6H). Additionally, immunocytochemistry of *Tet3KO* NE cultures showed a significant reduction in the percentage of Nestin^+^ and βIII-tubulin^*+*^ cells compared to wild-type iPSCs (Figure 6C). These findings suggest that TET3 may play a critical role in the acquisition of naive pluripotency during the reprogramming of adult NSCs and is essential for proper neural differentiation.

To investigate whether the loss of differentiation capacity in *Tet3-deficient* iPSCs correlates with altered methylation levels at ICRs, and thus with lack of pluripotency, we performed bisulfite conversion followed by the pyrosequencing of the Snrpn-DMR in wild-type and *Tet3KO* NSCs and derived iPSCs. *Tet3*KO iPSCs showed a very similar hypomethylation pattern compared to NSCs, as described for wild-type cells (Figure 6D), indicating that TET3 does not regulate the methylation status of this specific DMR. Notably, a significant loss of methylation was observed at the IG-DMR after the reprogramming of *Tet3KO* NSCs into iPSCs (Figure 6D). These data demonstrate that TET3 is necessary for maintaining IG-DMR methylation levels during the reprogramming process.

To elucidate the specific role of TET3 in safeguarding IG-DMR methylation during reprogramming, we performed an *in silico* analysis of TET3 binding using chromatin immunoprecipitation followed by deep sequencing (ChIP-seq) data from the ChIP-Atlas database in ESCs.^48^ The analysis showed that TET3 does not directly bind to the IG-DMR, but identified some factors with high binding occurrences at the IG-DMR (Figures S7A and S7B). One of them was TRIM28, a protein that interacts with the KRAB domain zinc finger protein ZFP57, which in turn serves as a scaffold for the recruitment of multiple epigenetic factors.^49^ Consistent with this, our analysis also identified ZFP57 as an IG-DMR-binding protein (Figure S7A). Additionally, OCT4, which has been implicated in driving ICR hypomethylation in post-implantation embryos, was also found to bind the IG-DMR (Figure S7A). Our analysis further showed TET3 binding to the *Trim28* and *Oct4* promoters in ESCs (Figure S7B). Interestingly, qPCR analysis revealed the upregulation of *Trim28*, *Oct4* and *Zfp57* in wild-type iPSCs compared to NSCs (Figures S7C and 6E).^50^ Notably, *Trim28* upregulation was not observed in *Tet3KO* iPSCs (Figure 6E), while *Oct4* levels were significantly higher in *Tet3KO* iPSCs than in wild-type iPSCs (Figure 6E) and remained elevated even after differentiation into neuroectoderm (Figure S7D). *Zfp57* expression was unaffected in *Tet3KO* iPSCs (Figure 6E).

These findings led us to hypothesize that TET3 may contribute to the maintenance of IG-DMR methylation in iPSCs by transcriptionally regulating key epigenetic and pluripotency-associated factors, specifically *Trim28* and *Oct4*. To investigate this, we performed chromatin immunoprecipitation followed by quantitative PCR (ChIP-qPCR) in wild-type adult NSCs to assess whether TET3 directly binds to the promoter regions of these genes (Figure 6F). TET3 enrichment was detected at both the *Trim28* and *Oct4* promoters (Figure 6G), with binding levels comparable to those observed at the *Snrpn* promoter, a known TET3 target.^12^ No significant enrichment was observed at a distal intergenic region, confirming the specificity of TET3 interaction (Figure 6G). These results suggest that TET3 may indirectly safeguard the methylation status of the IG-DMR by regulating the transcription of *Trim28* and *Oct4*, which are themselves involved in chromatin remodeling and imprinting stability. Importantly, the analysis of methylation levels in human pluripotent cells showed that the IG-DMR maintains hypermethylation after differentiation into NSCs (Figure S8A).^51^ Moreover, TRIM28 binds to the human IG-DMR (Figure S8B), suggesting the presence of a conserved mechanism of methylation maintenance at this locus also in humans. Taken together, these data support a non-canonical role for TET3 in protecting the IG-DMR from reprogramming-associated global hypomethylation, acting through the modulation of a regulatory axis essential for pluripotency maintenance.

## Discussion

Our study uncovers a previously undescribed mechanism by which the dioxygenase TET3 plays a pivotal role in maintaining DNA methylation levels during the reprogramming of adult NSCs into iPSCs. Reprogramming with the transcription factors *Oct4* and *Klf4* triggers extensive changes in gene expression and widespread DNA demethylation, including at ICRs. Notably, we found that the IG-DMR, the ICR regulating the imprinted *Dlk1-Dio3* cluster on mouse chromosome 12, is uniquely resistant to this global demethylation. This protection is critically dependent on TET3, which exerts its effects indirectly by regulating the transcription of *Trim28* and *Oct4*. Our model proposes that the TRIM28 protein binds to the IG-DMR and recruits DNMT enzymes to preserve its methylation during reprogramming. In the absence of TET3, *Trim28* is insufficiently expressed, leaving the IG-DMR vulnerable to demethylation. This permits OCT4 binding, which further promotes loss of methylation at this site. Crucially, TET3 deficiency not only disrupts methylation maintenance at the IG-DMR but also compromises the differentiation capacity of the resulting iPSCs. Together, these findings reveal an unexpected role for TET3 in safeguarding the epigenetic integrity of the *Dlk1-Dio3* cluster and ensuring the functional competence of pluripotent stem cells, underscoring its essential contribution to successful reprogramming and pluripotency maintenance.

DNA methylation at ICRs is essential not only for the establishment of genomic imprinting during development but also for its maintenance in somatic cells.^1^ Germline DMRs initiate monoallelic expression, while somatic DMRs help preserve this expression pattern throughout life. A growing body of literature highlights the critical role of imprinting dynamics also in the acquisition and maintenance of pluripotency.^20^^,^^21^^,^^22^^,^^23^^,^^24^ Reprogramming toward a naive pluripotent state recapitulates several epigenetic features of the early embryo, including global DNA demethylation at promoters of pluripotency genes that also affects ICRs.^14^^,^^24^ In line with this, we found that most ICRs across imprinted clusters are hypomethylated in iPSCs derived from adult NSCs, correlating with changes in the expression of multiple imprinted genes, suggesting that reprogramming actively reshapes the imprinting landscape.

Interestingly, the IG-DMR is an exception to this global trend. Despite the widespread loss of DNA methylation in iPSCs, this region retains its hypermethylated status already acquired in adult NSCs, which may reflect a previously unrecognized mechanism for maintaining imprinting fidelity in reprogrammed cells.^38^ Aberrant imprinting at the *Dlk1-Dio3* cluster has been linked to impaired developmental potential, supporting the hypothesis that maintaining hypermethylation at this locus may play a protective role in pluripotency.^16^^,^^23^ While previous work has demonstrated that deletions of specific regulatory elements within the IG-DMR result in imprinting loss in fibroblast-derived iPSCs, the potential consequences of these perturbations on pluripotency were not explored.^52^ In contrast, our study focuses on NSC-derived iPSCs that already exhibit a pre-existing biallelic hypermethylation at the *Dlk1-Dio3* locus, providing a unique model to test the impact of demethylation at this imprinted region.^38^ Upon treatment with the DNA methylation inhibitor 5-AZA, we observed a marked reduction in global 5mC levels as well as specific hypomethylation at the IG-DMR. Notably, this demethylation was accompanied by transcriptional changes indicative of compromised pluripotency, including a marked downregulation of *Nanog* and upregulation of *Nes*, a neural lineage marker. These results suggest that loss of methylation at the IG-DMR destabilizes the pluripotent state and predisposes cells toward lineage specification. Therefore, although some studies suggest the *Dlk1-Dio3* cluster is dispensable for reprogramming, our findings align with the notion that proper methylation at this site, rather than its transcriptional activity per se, correlates more robustly with iPSC quality and developmental competence.^26^^,^^27^^,^^53^^,^^54^

Many mechanistic aspects of how genomic imprinting is established, maintained, and subsequently erased during reprogramming remain unresolved. It is well known that DNA demethylation proceeds through one of the two distinct mechanisms: passive loss of 5mC during DNA replication via the suppression of DNMT activity or active demethylation by TET enzymes.^55^ Active demethylation is initiated through the progressive oxidation of 5mC to 5hmC, after which demethylation is achieved.^56^^,^^57^ Previous studies have suggested that TET proteins may play a role in active demethylation at some imprinted loci.^58^^,^^59^ However, the individual contribution of different TET has not been dissected, and effects are variable depending on the ICR and cellular context.^58^^,^^59^ While *Tet1* and *Tet2* expression are induced during iPSC generation, a higher level of expression of *Tet3* in adult NSCs is observed.^12^ Here we address the potential role of TET3 in preserving IG-DMR methylation during iPSCs reprogramming by using a murine model with conditional *Tet3* deletion in *Gfap*-expressing cells. Our results confirm that *Tet3*-deficient NSCs showed incomplete reprogramming into iPSCs, with the reduced upregulation of *Nanog* and diminished downregulation of *Nes*, indicating defective pluripotency acquisition. *Tet3KO* iPSCs maintain their ability to differentiate into all three germ layers; however, when directed toward neuroectoderm, they also fail to achieve proper neural differentiation, suggesting a critical role for TET3 for the acquisition of naive pluripotency during reprogramming.

Further methylation analysis using the pyrosequencing of the Snrpn-DMR revealed no significant differences in methylation between wild type and *Tet3KO* iPSCs. However, *Tet3KO* iPSCs exhibited a marked loss of methylation at the IG-DMR, suggesting that high levels of TET3 expression are particularly important at the onset of reprogramming, when the genome undergoes a wave of global demethylation. Our study also shows that TET3 plays a protective role at this critical stage, safeguarding the methylation of the IG-DMR. In contrast, this early protection does not occur in *Tet3KO* iPSCs, and consequently, the IG-DMR loses its methylation. Once the protective mechanism has been established, reduced levels of TET3 appear to be sufficient to maintain IG-DMR methylation throughout subsequent stages of reprogramming.

*In silico* analysis of TET3 binding revealed that TET3 does not directly bind to the IG-DMR, but it likely regulates genes encoding proteins that bind this region. In line with this, we identify TRIM28 and OCT4 as key proteins with significant binding activity at the IG-DMR in iPSCs. These two proteins have been reported to interact to protect methylation imprints at the IG-DMR from both active and passive demethylation during preimplantation development.^60^ Gene expression analysis confirmed that genes encoding for these two proteins are upregulated in iPSCs, whereas *Trim28* expression was not induced in Tet3KO iPSCs, indicating a direct regulation of this gene by TET3. Finally, we demonstrate that OCT4, a key pluripotency factor known to promote the hypomethylation of ICRs in post-implantation embryos, also binds the IG-DMR. *Oct4* levels were upregulated during reprogramming and were significantly higher in *Tet3*-deficient compared to wild-type iPSCs, suggesting that TET3 also regulates *Oct4* expression.^50^ TET3 binding at the *Trim28* and *Oct4* promoters, as revealed by ChIP analysis in adult NSCs, confirms its regulatory role over these genes, which in turn have antagonistic roles in controlling methylation at the IG-DMR.

Altogether, our work reveals that the epigenetic reprogramming of ICRs is not uniform and that locus-specific protection mechanisms, such as those involving TET3 and TRIM28 at the IG-DMR, are crucial to preserve imprinting stability. These insights not only reconcile contradictory findings about the role of the *Dlk1-Dio3* locus but also highlight the need to evaluate imprinting maintenance at the DNA methylation level, rather than solely through gene expression. As imprinting defects in iPSCs have been shown to persist after differentiation and compromise developmental potential, identifying regions resistant to demethylation and understanding their regulatory mechanisms will be critical for optimizing reprogramming protocols and improving the functional integrity of iPSC-derived cells in therapeutic contexts.^20^^,^^24^

In conclusion, our study demonstrates that iPSCs derived from adult NSCs retain a hypermethylated state at the IG-DMR, a feature that originates from their somatic precursor rather than arising *de novo* during reprogramming. While loss of imprinting is often interpreted as a defect incompatible with full pluripotency, our findings suggest that changes at specific ICRs, such as the persistent hypermethylation at the IG-DMR, may constitute an essential component of the epigenetic reorganization required for successful reprogramming. Importantly, we uncover a non-canonical role for TET3 in safeguarding methylation at the IG-DMR through the transcriptional regulation of *Trim28* and repression of *Oct4*. In this context, TET3 indirectly promotes the TRIM28-mediated recruitment of DNMTs to the IG-DMR while simultaneously preventing OCT4 binding, which would otherwise facilitate its demethylation. Loss of TET3 disrupts this regulatory axis, leading to the hypomethylation of the IG-DMR and impairing the maintenance of the pluripotent state. These findings are further supported by our functional assays, showing that the artificial demethylation of the IG-DMR via 5-azacytidine is sufficient to compromise core pluripotency markers and promote lineage-specific gene expression. Altogether, our work identifies the TET3-TRIM28-OCT4 axis as a critical mechanism for imprint maintenance and highlights the importance of epigenetic stability at the *Dlk1-Dio3* locus for sustaining the molecular identity of iPSCs. These insights contribute to a refined understanding of imprinting dynamics during reprogramming and offer a framework for improving the fidelity and functional potential of iPSCs in regenerative applications.

### Limitations of the study

Our study uncovers a non-canonical role of TET3 in maintaining IG-DMR methylation during the reprogramming of adult NSCs into iPSCs. However, several limitations should be noted. First, all experiments were conducted in a murine system, and although *in silico* analyses in human datasets suggest a similar regulatory mechanism, we did not experimentally validate this in human cells. Second, while we analyzed multiple ICRs, our work primarily focused on germline-derived regions, and the regulation of somatic ICRs was not extensively addressed. Third, although our data support an indirect role for TET3 through the transcriptional regulation of *Trim28* and *Oct4*, we cannot exclude the contribution of additional pathways or cofactors. Finally, although our functional assays link IG-DMR methylation to pluripotency and differentiation capacity, further *in vivo* studies will be necessary to confirm the developmental consequences of TET3 deficiency.

## Resource availability

### Lead contact

Further information and requests for resources and reagents should be directed to and will be fulfilled by the lead contact, Sacri R. Ferron (sacramento.rodriguez@uv.es).

### Materials availability

Induced pluripotent stem cell (iPSC) lines generated in this study are available from the lead contact upon the completion of a material transfer agreement (MTA).

### Data and code availability

Data: Raw and processed sequencing data generated in this study have been deposited in the Gene Expression Omnibus (GEO) under the following accession numbers: GEO: GSE282749 (methylation data) and GEO: GSE282748 (RNA-seq data). Processed RNA-seq raw counts and scaled MeDIP-seq signal tracks are available in the corresponding GEO entries. Accession numbers are listed in the key resources table. All other data supporting the findings of this study are available within the main text and its supplemental information.

Code: This study did not generate original code. All software and computational tools used for data processing and analysis are cited in the method details section and summarized in Table S9. Non-default parameters used for RNA-seq and MeDIP-seq analyses are specified in the respective analysis subsections.

Other: All materials and reagents used in this study are listed in the key resources table, including iPSC lines generated in this work and their corresponding identifiers. Any additional information required to reanalyze the data reported in this article is available from the lead contact upon reasonable request.

## Acknowledgments

We firstly would like to thank Dr. Isabel Fariñas, Dr. Anne Ferguson-Smith and Dr. Ángel Raya and their groups for technical support and discussion of the data. This work was supported by grants from Ministerio de Ciencia e Innovación/AEI (PID2019-110045GB-I00, PID2022-142734OB-I00 and EUR2023-143479), 10.13039/501100003359Generalitat Valenciana (AICO/2020/367) and 10.13039/100007406Fundación BBVA to S.R.F. L.L.C. (PRE2020-094137) and J.D.M. (PRE2022-000680) were funded by the Spanish Formación de Personal Investigador (FPI) fellowship program E.J.V. was funded by the Spanish Formación de Profesorado Universitario (FPU) fellowship program (FPU20/00795). A.L.U. was funded by the 10.13039/501100003359Generalitat Valenciana fellowship program (ACIF/2016/381). Open Access funding was provided by the Ministerio de Ciencia e Innovación.

## Author contributions

L.L.C., E.J.V., A.L.U., R.M.L., J.D.M., and A.L.P. carried out most of the experiments. L.L.C. performed gene expression analysis, ChIP experiments, and statistical analysis. E.J.V. performed DNA methylation assays, helped by M.I., and A.L.U. R.M.L., and J.D.M. performed NSC reprogramming. A.L.P. contributed to developing the reprogramming protocol. J.P. performed the bioinformatic analysis of transcriptome and methylome data. E.J.R. helped with MeDIP-seq bioinformatic analysis. S.R.F. initiated, designed, and led the study and wrote the article. All authors contributed to experimental design, data analysis, discussion, and writing of the article.

## Declaration of interests

The authors declare no competing financial interests.

## Declaration of generative AI and AI-assisted technologies in the writing process

During the preparation of this work, the authors used ChatGPT in order to improve the language and ensure grammatical accuracy. After using this tool, the authors reviewed and edited the content as needed and take full responsibility for the content of the publication.

## STAR★Methods

### Key resources table

**Table:** 

REAGENT or RESOURCE	SOURCE	IDENTIFIER
**Antibodies (see**Tables S3**and**S4**)**		

5mC	Diagenode	Cat#C15200006; RRID: AB_3343984
α-fetoprotein	R&D	Cat#mab1368; RRID: AB_357658
βIII-tubulin	Covance	Cat#PRB-435P; RRID: AB_291637
Brachyury	Santa Cruz	Cat#sc-17743; RRID: AB_634980
GATA-4	Santa Cruz	Cat#sc-1237; RRID: AB_2108747
IgG	Santa Cruz	Cat#sc-2027; RRID: AB_737197
NANOG	Reprocell	Cat#RCAB002PF; RRID: AB_2616320
Nestin	Hybridoma bank	Cat#rat-401; RRID: AB_2235915
OCT-4	Santa Cruz	Cat#sc-5279; RRID: AB_628051
OLIG2	Millipore	Cat#AB9610; RRID: AB_570666
α-Smooth Muscle Actin (α-SMA)	Abcam	Cat#ab18147; RRID: AB_444285
SOX2	R&D Systems	Cat#AF2018; RRID: AB_355110
SSEA1	Stemgent	Cat#09-0067; RRID: AB_1862373
TET3	Millipore	Cat#ABE290
Alexa Fluor® 488 Donkey Anti-Mouse	Molecular Probes	Cat#A-21202; RRID: AB_141607
Alexa Fluor® 488 Donkey Anti-Rabbit	Jackson ImmunoResearch	Cat#711-547-003; RRID: AB_2340620
Alexa Fluor® 647 Donkey Anti-Rabbit	Jackson ImmunoResearch	Cat#711-607-003; RRID: AB_2340626
Cy3-Donkey Anti-Rabbit	Jackson ImmunoResearch	Cat#711-165-152; RRID: AB_2307443
Cy3-Donkey Anti-Goat	Jackson ImmunoResearch	Cat#705-166-147; RRID: AB_2340413
Cy3-Donkey Anti-Mouse	Jackson ImmunoResearch	Cat#715-165-151; RRID: AB_2315777

**Bacterial and virus strains**		

pMXs-*Oct4*	Addgene	Cat#13366; RRID: Addgene_13366
pMXs-*Klf4*	Addgene	Cat#13370; RRID: Addgene_13370
pMXs-*mCherry*	This paper	N/A

**Chemicals, peptides, and recombinant proteins**		

Earle’s balanced salt solution	Gibco	Cat#24010-043
Papain	Worthington	Cat#LS003119
L-cystein	Sigma-Aldrich	Cat#C8277
EDTA	Sigma-Aldrich	Cat#E6511
Trypsin/EDTA	Gibco	Cat#25300054
Dulbecco’s modified Eagle’s medium (DMEM)	Gibco	Cat#11320-074
L-glutamine	Gibco	Cat#25030-024
D-Glucose	Sigma-Aldrich	Cat#G7021
Putrescine	Sigma-Aldrich	Cat#P7505
Progesterone	Sigma-Aldrich	Cat#P6149
Sodium selenite	Sigma-Aldrich	Cat#S9133
Insulin	Sigma-Aldrich	Cat#I6634
Apo-transferrin	Sigma-Aldrich	Cat#T2252
Heparin	Sigma-Aldrich	Cat#H3149
Epidermal growth factor	Gibco	Cat#53003-018
Fibroblast growth factor	Sigma-Aldrich	Cat#F0291
Opti-MEM	Gibco	Cat#31985062
Polyethylenimine	Polysciences	Cat#23966
Foetal bovine serum (for ESCs)	Capricorn	Cat#FBS-12A
Foetal bovine serum (for NSCs)	Biowest	Cat#S181B-500
Puromycin	Sigma-Aldrich	Cat#P8833
Blasticidin	Sigma-Aldrich	Cat#SBR00022
Dimethyl sulfoxide (DMSO)	PanReacAppliChem	Cat#A3672
Polybrene	Sigma-Aldrich	Cat#TR1003
Mitomycin	Sigma-Aldrich	Cat#10107409001
Gelatin	Sigma-Aldrich	Cat#G1890
Accutase	Sigma-Aldrich	Cat#A6964
LIF	This paper	N/A
Glasgow Minimum Essential Medium	Gibco	Cat#11710035
β-mercaptoethanol	Sigma-Aldrich	Cat#M6250
Sodium pyruvate	Gibco	Cat#11360070
B27 supplement	Gibco	Cat#A3582801
MEK inhibitor	Millipore	Cat#PZ0162
GSK3 inhibitor	Millipore	Cat#CHIR99021
Accumax	Sigma-Aldrich	Cat#A7089
Poly (2-HEMA) solution	Sigma-Aldrich	Cat#P3932
Neurobasal	Gibco	Cat#21103049
DMEM High glucose	Biowest	Cat#L0101
DMEM-GlutaMAX™	Gibco	Cat#10565018
GlutaMAX™	Gibco	Cat#35050061
BSA	Sigma-Aldrich	Cat#B4287
Matrigel	Corning	Cat#356234
KarioMAX Colcemid	Gibco	Cat#10116784
Leishman Staining	Sigma-Aldrich	Cat#L6254
Dimethylformamide	Sigma-Aldrich	Cat#227056
Fast red salt	Sigma-Aldrich	Cat#368881
Naphtol phosphate	Sigma-Aldrich	Cat#N4875
TRI reagent	Sigma-Aldrich	Cat#93289
HotStar Taq polymerase	Qiagen	Cat#203203
Streptavidin Sepharose High Performance	GE Healthcare	Cat#17-5113-01
RNase A	Roche	Cat#10109142001
Proteinase K	Roche	Cat#03115879001
Dynabeads M-280 sheep anti-mouse IgG	Invitrogen	Cat#11201D
Dynabeads	Invitrogen	Cat#10003D
5-Aza-2′-deoxycytidine (5-AZA)	Sigma-Aldrich	Cat#A3656
Paraformaldehyde (PFA)	Sigma-Aldrich	Cat#16005

**Critical commercial assays**		

RNAeasy Mini Kit	Qiagen	Cat#74104
RevertAid H Minus First Strand cDNA Synthesis kit	Thermo Scientific	Cat#K1622
TB Green Premix ExTaq	Takara	Cat#RR420A
TaqMan Fast Advanced Master Mix	Applied Biosystems™	Cat#4444963
Illumina TruSeq stranded mRNA Sample Preparation Kit v2	Illumina	Cat#20020594
DNeasy Blood and Tissue Kit	Qiagen	Cat#69504
NEBNext Ultra™ II FS DNA Library Prep Kit for Illumina	New England Biolabs	Cat#E7805S
MinElute PCR purification kit	Qiagen	Cat#28004
EZ DNA Methylation-GoldTM kit	Zymo research	Cat#D5005
PyroMark® Gold Q96 Reagents	Qiagen	Cat#972804

**Deposited data**		

RNA-seq raw and processed data	This paper	GEO: GSE282748
MeDIP-seq raw and processed data	This paper	GEO: GSE282749
Mouse reference genome UCSC mm10/GRCm38	Genome Research Consortium	https://hgdownload.soe.ucsc.edu/goldenPath/mm10/bigZips/; RRID: SCR_005780
Gencode reference transcriptome vM23	Gencode	https://ftp.ebi.ac.uk/pub/databases/gencode/Gencode_mouse/release_M23/; RRID: SCR_014966
ESC MeDIP-seq data	Cernilogar et al.^61^	GEO: GSE116262
ESC MeDIP-seq data	Chang et al.^62^	GEO: GSE36294
ESC MeDIP-seq data	Neri et al.^63^	GEO: GSE44644
Human WGBS data	Yan et al.^51^	GEO: GSE145964

**Experimental models: Cell lines**		

Mouse fibroblast SNL	Cell biolabs	CBA-316; RRID: CVCL_K227
Embryonic Stem Cell line E14Tg2a	ATCC	CRL-1821; RRID: CVCL_9108
PlatinumE (Plat-E) retroviral packing cells	Cell Biolabs	RV-101; RRID: CVCL_B488
Mouse Neural Stem Cells (NSCs)	Primary cultures (this paper)	N/A
Induced pluripotent Stem Cells (iPSCs)	Derived from NSCs (this paper)	N/A

**Experimental models: Organisms/strains**		

C57BL/6	The Jackson Laboratory	RRID:IMSR_JAX:000664
*Nude* (NU/J)	The Jackson Laboratory	RRID:IMSR_JAX:002019
6.Cg-Tg(Gfap-cre)73.12Mvs/J	The Jackson Laboratory	RRID:IMSR_JAX:012886
*Tet3*^*loxp/loxp*^	Taconic Biosciences GmbH	BSRC0002

**Oligonucleotides**		

TaqMan probes for gene expression	This paper (see Table S5)	Applied Biosystems
Primers for SYBR-green	This paper (see Table S6)	N/A
Primers to test MeDIP-seq specificity and enrichment	This paper (see Table S7)	N/A
Primers for pyrosequencing	This paper (see Table S8)	N/A

**Software and algorithms**		

Software and tools used for bioinformatic analyses	See Table S9	N/A

### Experimental model and study participant details

#### Animals and *in vivo* manipulations

The experiments were conducted in 2- to 4-month-old mice. For wild-type condition, C57BL/6 mice were used. For the teratoma formation assays, female homozygous immunosuppressed *Nude* (NU/J) mice obtained from the Jackson Laboratory were used. Littermates of the same sex were randomly assigned to experimental groups. For *Tet3* deletion in GFAP^+^ NSCs, heterozygous GFAP-cre transgenic animals (6.Cg-Tg(Gfap-cre)73.12Mvs/J) from the Jackson Laboratory were bred to *Tet3*^*loxp/loxp*^.^12^ Expression of Cre-recombinase under the *Gfap* promoter results in a deletion of exon 5 of *Tet3* gene, causing a frame-shift from exon 6 and a premature stop codon in exon 7 of the gene. Animals were genotyped by PCR analysis of DNA as described and littermates lacking *GFAP-Cre* were used as control mice.^12^ All mice were maintained on a C57BL/6 background and in a 12 h light/dark cycle with free access to food and water *ad libitum* and according to the Animal Care and Ethics committee of the University of Valencia.

#### Neurosphere cultures

Adult 2- to 4-month-old female mice were euthanized by cervical dislocation. To initiate each independent culture, the brains of two different animals were dissected and the regions containing the SVZ were isolated from each hemisphere and washed in Earle’s balanced salt solution (EBSS; Gibco). Tissues were transferred to EBSS containing 12 U mL^-1^ papain (Worthington), 0.2 mg mL^-1^ L-cystein (Sigma-Aldrich), 0.2 mg mL^-1^ EDTA (Sigma-Aldrich) and incubated for 20 min at 37°C. Tissue was then rinsed in EBSS, transferred to Dulbecco’s modified Eagle’s medium (DMEM)/F12 medium (1:1 v/v; Gibco) and carefully triturated with a fire-polished Pasteur pipette to a single cell suspension. Isolated cells were collected by centrifugation, resuspended and cultured in NSCs medium: DMEM/F12 medium containing 2 mM L-glutamine (Gibco), 0.6% D-glucose (Sigma-Aldrich), 9.6 μg mL^-1^ putrescine (Sigma-Aldrich), 6.3 ng mL^-1^ progesterone (Sigma-Aldrich), 5.2 ng mL^-1^ sodium selenite (Sigma-Aldrich), 0.025 mg mL^-1^ insulin (Sigma-Aldrich), 0.1 mg mL^-1^ apo-transferrin (Sigma-Aldrich), 4 mg mL^-1^ bovine serum albumin (BSA), 2 μg mL^-1^ heparin (sodium salt, grade II; Sigma-Aldrich) supplemented with 20 ng mL^-1^ epidermal growth factor (EGF; Gibco) and 10 ng mL^-1^ fibroblast growth factor (FGF; Sigma-Aldrich).^64^ Neurospheres were allowed to develop for 6 days in a 95% air-5% CO_2_ humidified atmosphere at 37°C. For culture expansion, neurospheres were collected, disaggregated using Accutase® (Sigma-Aldrich) and plated at a relatively high density (75 cell/μL) in NSCs medium.

### Method details

#### Reprogramming of adult NSCs into iPSCs

Adult NSCs derived from females were used as donor cells to generate iPSCs to avoid the previously reported sex-dependent genomic imprinting instability.^20^ To generate iPSCs from adult NSCs, exogenous *Oct4* together with *Klf4* (2F) were used for reprogramming as previously described.^17^ To produce retroviruses expressing *Oct4* and *Klf4*, PlatinumE (Plat-E) retroviral packing cells (RV-101, Cell Biolabs) were transfected with a plasmid solution containing 1 mL of Opti-MEM™ (Gibco), 60 μL of 1mg mL^-1^ polyethylenimine (PEI, Polysciences) and 20 μg of the retroviral vectors pMXs-*Oct4* (#13366, Addgene), pMXs-*Klf4* (#13370, Addgene) and pMXs-*mCherry* (pMX-2A-CH, designed and kindly provided by Dr. Jose Manuel Torres). After 24 hours, Plat-E culture medium (high glucose DMEM containing 10% foetal bovine serum FBS, 2 mM L-glutamine, 1 μg mL^-1^ Puromycin and 10 μg mL^-1^ Blasticidin) was replaced by NSCs medium. Transfection efficiency was checked by mCherry fluorescence in Plat-E cells (Figure S1A). The day after, retrovirus-containing supernatants were collected and filtered with a 0.45 μm nitrocellulose filter. Neurospheres were grown for two days and then transduced with a mixture of these supernatant (SN) as follows (volume *per* plate): 3 mL of *Oct4* SN, 3 mL of *Klf4* SN, 1 mL of *mCherry* SN and 3 ml of fresh NSCs complete medium. A control of infection was made with a mixture containing 7 mL of *mCherry* retrovirus containing medium and 3 mL of fresh complete medium. In order to enhance the efficiency of retroviral infection, retrovirus mixture was supplemented with 4 μg mL^-1^ of polybrene (hexadimethrine bromide, Sigma-Aldrich). NSCs were then incubated for 14-18 hours at 37°C in a humidified incubator. Infected NSC medium was then replaced with fresh complete medium and neurospheres were allowed to develop for 5 days (Figure 1B). The mouse fibroblast cell line SNL (CBA-316, Cell Biolabs) was used as feeder cells during the reprogramming process. SNL feeder cells were first mitotically inactivated by treatment with 4 μg mL^-1^ of Mitomycin C (Sigma) for 2-4 hours. Plates were treated with 0.1% of gelatin (Sigma-Aldrich) at 37°C for at least 20 min and then mitomyzed SNLs were plated at high density (2.5x10^6^ cell/plate) in gelatine-treated plates (day 7). Five days after transduction, neurospheres were dissociated with Accutase® (Sigma) and 1.5x10^5^ of infected NSCs were re-plated on SNL feeder cells with ESC/LIF medium: Glasgow Minimum Essential Medium (GMEM, Gibco) containing 15% FBS, 2 mM L-glutamine, 1 mM Sodium pyruvate (Gibco), 0,1 mM β-mercaptoethanol (Sigma-Aldrich) and 1 μM LIF. ESC/LIF medium was changed every other day until Stage-Specific Embryonic Antigen-1 (SSEA-1; also known as CD15) positive colonies appeared (pre-iPSCs), checked by staining with StainAlive SSEA-1 Antibody (DyLight 488) (Stemgent®, 1:100 dilution) (Figures 1F and S1A). ESC/LIF medium was replaced with 2i/LIF Neurobasal medium containing B27 supplement, 2 mM L-glutamine, 1 mM Sodium pyruvate, 1 mg mL^-1^ apo-transferrin, 50 μM Insulin, 16 μg mL^-1^ Putrescine, 60 ng mL^-1^ Progesterone, 0.3 μM Sodium selenite, 50 μg mL^-1^ BSA, 1 μM LIF, 1 μM iMEK (Millipore, PD03259) and 3 μM iGSK3 (Millipore, CHIR99021), which is a serum-free medium based on dual inhibition (2i) of mitogen-activated protein kinase (MAPK) signalling and glycogen synthase kinase-3 (GSK3) combined with LIF.^32^ 2i/LIF medium was changed every two days until well-defined iPSCs colonies appeared (Figure 1B). To establish and expand clonal lines of iPSCs, individual colonies were isolated and plated on gelatine treated plates with 2i/LIF medium. For 5-AZA assays, three independent iPSCs cultures growth in 2i/LIF medium were treated with vehicle (DMSO) or 10 μM 5-AZA for three days. The embryonic stem cell (ESC) line E14Tg2a was used as a pluripotency positive control in the different experiments. ESCs were cultured on gelatine-treated plates and 2 days after plating, cells were treated with Trypsin/EDTA and re-plated following a dilution of 1:5 in ESC/LIF medium.

#### Embryoid bodies assays

Embryoid bodies were obtained using the “*hanging drops*” method. iPSCs were treated with Accumax® (Sigma) and resuspended in EB medium: GMEM containing 10% FBS, 2 mM L-glutamine and 1 mM Sodium pyruvate. Several rows of 20 μL drops of a cell suspension at 30 cells/ μL were plated using a multichannel pipet (Figure 2A). Plates were incubated upside-down for 3 days at 37°C in a 5% CO_2_ humidified incubator. Plates were then inverted and EB medium was added. To avoid EB attachment plates were previously treated with 0.4% poly (2-HEMA) solution (Sigma-Aldrich) prepared in Ethanol:Acetone (1:1). EBs were incubated for 4 more days and then plated on gelatine-treated plates for 3 more days before analysis (Figure 2A).

#### Neuroectoderm differentiation

Neuroectoderm (NE) differentiation was performed as previously described.^65^ Briefly, iPSCs were treated with Accumax® and resuspended in NE culture medium: DMEM/F12 GlutaMAX™ (containing 25 μg/mL insulin, 100 μg mL^-1^ apo-transferrin, 6 ng mL^-1^ progesterone, 16 μg mL^-1^ putrescine, 30 nM sodium selenite, and 50 μg mL^-1^ BSA (Sigma)) and Neurobasal (containing 2% B27, 2 mM GlutaMAX™, 0.1% 2-mercaptoethanol, and 1.45% sterile D-glucose) (Figure 6C). Cells were counted and plated at a density of 1.5 x10^4^ cells/cm^2^ in gelatine pre-treated plates (Figure 6C). Cultures were maintained in NE culture medium and the medium replenished every other day for a week (Figure 6C).

#### Teratoma formation and analysis

To evaluate the capacity of iPSCs to generate teratomas, mouse iPSCs cultures were collected by treatment with Accumax®. iPSCs were washed in PBS and resuspended in PBS supplemented with 30% Matrigel® (Corning®).^37^ Cells were kept on ice and drawn into a 1 mL syringe immediately before injection. Approximately 1.5×10^6^ cells resuspended in 200 μL of solution were injected in the dorsolateral area of the subcutaneous space on both sides of the mice back. Teratomas were allowed to develop for 15-20 days when the size of the teratomas was approximately 1.5-2 cm. Mice were sacrificed by cervical dislocation and teratomas were extracted for analysis. For teratoma analysis, samples were fixed in 4% paraformaldehyde (PFA) overnight at 4°C with shaking. Samples were embedded in paraffin and teratoma samples were serially sectioned into 7 μm sections using a microtome (Leica). Slices were stained with haematoxylin and eosin and cell types from the three embryonic layers were identified under the optic microscope (Nikon Eclipse Ni).

#### Karyotype of iPSCs

To perform the karyotype analysis, cell division was inhibited using 0.6 μg mL^-1^ of KarioMAX® Colcemid (Gibco) at 37°C. After 2 hours, culture medium was removed and 0.85% sodium citrate, previously warmed at 37°C, was added. A cell scraper was used to raise the cells. Cell suspension was transferred to a 15 mL conical tube and incubated at 37°C for 15 min. After that, 10 drops of cold Carnoy fixative (methanol-acetic acid, 3:1) were added to the suspension and softly mixed using a Pasteur pipette. Samples were washed several times with 5 ml of cold Carnoy solution and, after centrifugation (10 min, 300 *g*), pellets were resuspended in 2 drops of Carnoy fixative. Cells extensions were made in microscope slides followed by heat fixation. Samples were stained with Leishman's stain (Sigma). The number of chromosomes was determined under the optic microscope (Nikon Eclipse Ni).

#### Immunocytochemistry and alkaline phosphatase (AP) staining

NSCs, iPSCs and EBs were fixed for staining with 4% PFA in 0.1M PBS for 15 min and immunocytochemistry performed as previously described.^64^ Primary and secondary antibodies and dilutions used are listed in Tables S3 and S4 respectively. DAPI (1 μg mL^-1^) was used to counterstain DNA. Samples were photographed and analysed using an FV10i confocal microscope (Olympus). Alkaline phosphatase detection method was used in reprogrammed cells to check the presence of iPSCs after one month in 2i/LIF medium on *feeders*. Cells were fixed with cold methanol for 2 min and washed three times with 0.1 M Tris-HCl pH 8.5 buffer. Samples were incubated with the “*staining solution*” which contained 0.1 mg mL^-1^ naphtol phosphate (Sigma-Aldrich), 0.5% Dimethylformamide (Sigma-Aldrich) and 0.6 mg mL^-1^ Fast Red Salt (Sigma-Aldrich) in 0.1 M Tris-HCl pH 8.5. When red precipitate appeared, cells were washed with 0.1M Tris-HCl and distilled water. Finally, the different plates were photographed using a microscope-coupled camera.

#### Expression studies

RNAs were extracted with RNAeasy Mini Kit (Qiagen) including DNase treatment, following the manufacturer’s guidelines. For qPCR, 1 μg of total RNA was reverse transcribed using random primers and RevertAid H Minus First Strand cDNA Synthesis kit (Thermo Scientific), following standard procedures. Thermocycling was performed in a final volume of 10 μL, containing 4-10 ng of cDNA sample and the reverse transcribed RNA was amplified by PCR with appropriate Taqman probes (Table S5). qPCR was used to measure gene expression levels relative to *Gapdh*, which expression did not differ among the groups. qPCR reactions were performed in a Step One Plus cycler with Taqman Fast Advanced Master Mix (Applied Biosystems). In case of using SYBR green, thermocycling was also performed in a final volume of 10 μL, containing 4-10 ng of cDNA sample, 0.2 μM of each primer (Table S6) and SYBR® Premix ExTaq™ (Takara) according to the manufacture instructions, using ROX as a reference dye. A standard curve made up of doubling dilutions of pooled cDNA from the samples being assessed was run on each plate, and quantification was performed relative to the standard curve.

#### RNA-seq

RNA was isolated using TRI Reagent® (Sigma) following manufacturer’s instructions. Briefly, 1 mL of reagent was added per 5-10x10^6^ cells for lysis during 20 min at room temperature (RT). Then, 100 μL of chloroform was added to samples and incubated at RT for 10 min and centrifuged at 12,000*g* centrifugation at 4°C for 10 min. For RNA precipitation, aqueous phase was mixed with 500 μL of isopropanol and incubated for 5 min. Samples were centrifuged 8 min at 12,000*g* at 4°C. RNA pellet was washed in 1 mL of 75% ethanol, vortexed and centrifuged 5 min at 7500 *g* at 4°C. Then, RNA pellet was resuspended in RNase-free water and stored at -80°C until use. Library preparation and high-throughput sequencing were performed by the Central Service for Experimental Research (SCSIE) at the University of Valencia. Libraries were generated from triplicated biological samples per condition using the Illumina TruSeq stranded mRNA Sample Preparation Kit v2 following the manufacturer’s protocol and sequenced using Illumina NextSeq 500. Read quality was assessed with *FastQC*. Expression was quantified at gene level with *salmon*^66^ in pseudomapping mode, with automatic library detection (-l A) and sequence bias correction (--seqBias) using the Gencode release M23 as reference.^67^ Gene expression quantification was imported into R with package *tximeta*^68^ and differential expression analysis was performed with DESeq2.^69^ Gene ontology (GO) analysis was conducted with R package clusterProfiler.^70^ *Ggplot2* was used for visualizations and *dplyr*, *tibbl*e and *tidyr* packages for data wrangling.^71^ Heatmap visualizations were performed with Complex Heatmap package and scaled variance stabilized counts.^72^

#### MeDIP-seq

DNA was extracted with DNeasy Blood and Tissue Kit (Qiagen) following the manufacturer’s instructions. Samples were eluted in 100 μL of elution buffer and DNA concentration was measured using a Nanodrop 1000. MeDIP-seq protocol was adapted from Taiwo et al., 2012.^73^ For immunoprecipitation, 3 μg of DNA were sonicated to obtain 150-200 bp fragments. Sonication efficiency was checked by capillary electrophoresis (Bioanalyzer, Agilent). DNA libraries were prepared using NEBNext® Ultra™ II FS DNA Library Prep Kit for Illumina (New England Biolabs). For MeDIP, 1.5 μg of DNA was diluted in TE buffer (10 mM Tris-HCl, 1 mM EDTA, pH 7.5) and denatured for 10 min at 99°C. Non-specific interactions were blocked by adding 20 μL of 10x IP buffer (100 mM Na-Phosphate pH 7.0, 0.5% TritonX-100) and 100 μL of 5% skimmed milk buffer in 2 M NaCl. Then 2 μg of anti-5mC antibody (Diagenode) were added and incubated for 2 h at 4°C with rotation. In parallel, 11 μL per sample of Dynabeads® M-280 sheep anti-mouse IgG (Invitrogen) were blocked with 500 μL PBS-BSA (1 mg mL^-1^ BSA in 0.1 M PBS) for 2 hours at 4°C with rotation. After incubations, beads were collected in a magnetic rack, re-suspended in the original volume (11 μL) with 1x IP buffer (10 mM Na-Phosphate pH 7.0, 0.05% TritonX-100, 1 M NaCl) and added to the DNA samples, which were incubated overnight at 4°C with rotation. The day after, beads were collected using a magnetic rack and the supernatant (unbound fraction) was transferred to a fresh tube. Beads were washed three times with 500 μL of 1x IP buffer for 10 min with rotation at 4°C. After the final wash, bound and unbound fractions were treated with 0.3 mg/mL of Proteinase K (Roche) in digestion buffer (50 mM Tris-HCl pH 8.0, 10 mM EDTA, 0.5% SDS) and incubated at 55°C for 30 min on a shaking heating block. Samples were purified using MinElute PCR purification kit (Qiagen) and eluted in 10 μL of elution buffer. To calculate 5mC enrichment in the bound fraction, quantitative PCRs for unmethylated and methylated regions were done from bound and unbound fractions (Figure S4A). Enrichment should be at least 25x, specificity should be more than 95% and unmethylated recovery should be less than 1% (Figure S4A). Samples were sequenced in a HiSeq2000 (Illumina, Inc) instrument. Primers used to evaluate MeDIP efficiency and specificity are provided in Table S7.

MeDIP-seq reads were pre-processed using MEDUSA pipeline with default settings and the UCSC mm10 was used as the reference genome.^74^ Signal tracks were obtained with *Deeptools* suite, with the bamCoverage function, applying the following parameters: --scaleFactor X --binSize 1 --blackListFileName --minMappingQuality 20.^75^ The blacklist file was sourced from Boyle Lab Github repository.^76^ The scaling factor was determined using the normalization factor obtained from counting reads in 10 kb bins, followed by TMM normalization using the *edgeR::calcNormFactors* function.^77^ Biological replicates were combined using the *bigwigAverage* function from Deeptools, averaging the methylation signal. Read counting, CpG density normalization, and differential methylation analysis were performed using the *MEDIPS* R package.^78^ A sliding window of 100 bp was applied, and a minimum read depth of 10 across all samples was required for inclusion in the analysis. Differential methylation was modeled with the *edgeR* implementation in the *MEDIPS* package, using TMM normalization and multiple testing correction via the Benjamini-Hochberg method.^77^ Principal component analyisis (PCA) was performed using the *DESeq2* package.^69^ To ensure robust signal detection, we retained the top 5% percentile windows with the highest signal and discarded those that did not overlap a CpG island or a CpG shore (obtained from UCSC mm10 repository). Adjacent windows were merged by calculating the harmonic mean of p-values using the extraChIPs::mergeByHMP function with the following parameters: merge_within = 1, p_adj_method = fdr, alpha = 0.01. Finally, windows were annotated to genes using the *ChIPseeker* package.^79^ For ICR analysis, we used previously defined ICRs, completed with additional somatic ICR locations defined in our laboratory (Tables S1 and S2).^44^ We extracted windows overlapping ICR genomic regions prior to CpG filtering using the R package *plyranges* and the *join_overlap_inner* function.^80^ Adjacent windows were merged, as described previously, with the merge_within parameter set to 300. Gene ontology (GO) analysis, data wrangling, and visualization were performed with the packages. Metagenes were generated by combining the *ComputeMatrix* reference-point and *plotProfile* functions from the *Deeptools* suite, which represent the methylation signal at nucleotide resolution (--bin Size 1) within a -5000/1500 bp window (-a 1500, -b 5000) around the TSS (--reference Point TSS). Signal tracks were visualized using SparK, with averaged the scaled methylation signal across biological replicates over the selected ICR genomic regions.^81^ Chromatin states were obtained from Vu and Ernst 2023 and overlapped using *plyranges* and *join_overlap_inner* function.^43^^,^^80^ ESC MeDIP-seq data was accessed from Gene Expression Omnibus (GEO), corresponding to GEO: GSE116262, GSE36294, and GSE44644,^61^^,^^62^^,^^63^ and analyzed as explained above.

#### DNA methylation analysis by pyrosequencing

DNA methylation level was quantified using bisulfite conversion and pyrosequencing. The DNA was bisulfite-converted using EZ DNA Methylation-GoldTM kit (Zymo research) in accordance with the manufacture's protocol. Specifically, for the different DMRs, bisulfite-converted DNA was amplified by PCR with specific primer pairs (Table S8). PCRs were carried out in 20 μL, with 2U HotStar Taq polymerase (Qiagen), PCR Buffer 10x (Qiagen), 0.2 mM dNTPs and 400 mM primers. PCR conditions were: 96°C for 5 min, followed by 39 cycles of 94°C for 30 s, 54°C for 30 s and 72°C for 1 min. For pyrosequencing analysis, a biotin-labelled primer was used to purify the final PCR product using sepharose beads. The PCR product was bound to Streptavidin Sepharose High Performance (GE Healthcare), purified, washed with 70% ethanol, denatured with 0.2 N NaOH and washed again with 10 mM Tris-acetate. Pyrosequencing primer (400 mM) was then annealed to the purified single-stranded PCR product and pyrosequencing was performed using the PyroMark Q96MD pyrosequencing system using PyroMark® Gold Q96 Reagents (Qiagen).

#### Chromatin immunoprecipitation (ChIP)

ChIP assays were performed using 1 × 10^7^ wild-type NSCs isolated from the adult SVZ. Cells were crosslinked with formaldehyde, and chromatin was extracted and sheared to an average fragment size of 200–500 bp using a Bioruptor sonicator (Diagenode, UCD-200). Sheared chromatin was pre-cleared with 20 μL of protein G magnetic beads (Dynabeads®, Invitrogen) for 45 minutes at 4°C. Pre-cleared samples were then incubated overnight at 4°C with 10 μg of anti-TET3 antibody or non-immune rabbit IgG (see Table S3 for antibody details). Immunocomplexes were captured using 20 μL of Dynabeads for 3 hours at 4°C. Five percent of the chromatin was reserved as input control before immunoprecipitation. Beads were then washed extensively, and the protein–DNA complexes were eluted and reverse-crosslinked overnight at 65°C in the presence of RNase A. Proteins were digested with Proteinase K for 2 hours at 45°C. DNA was purified using the Qiagen MinElute PCR Purification Kit, according to the manufacturer’s instructions. ChIP-enriched DNA was quantified by qPCR using SYBR Green chemistry and primer sets listed in Table S6. Pull-downs using rabbit IgG served as negative controls to assess non-specific enrichment. Quantification of ChIP recovery was performed using the percent input method, calculated with the following formula:

$$\%ChIP\;signal/input=2^{\left(CtInput-\log_{2}\left[dilution\;factor\right]-CtChIP\right)}x100$$

This value reflects the proportion of total chromatin specifically immunoprecipitated relative to the input. Representative uncropped scans of PCR products are shown in Figure S9. To characterize the protein binding landscape at ICRs in ESCs, we used the ChIP-Atlas 3.0 database (mouse genome mm10), using the 'Transcription Factors and Others' category, with all antibodies and all cell types included in the track selection.^48^

### Quantification and statistical analysis

All statistical tests were performed using the GraphPad Prism Software, version 7.00 for Windows. Data were first tested for normality using Shapiro-Wilk test. The significance of differences between groups was evaluated using appropriate statistical tests for each comparison. For data that passed normality tests: a t-test was used when comparing only two groups (applying Welch’s correction in case the standard deviation of groups is different); and one-way ANOVA followed by Šídák's post-hoc test was applied for comparing three or more groups. For data groups that did not pass normality: Mann–Whitney or unpaired Wilcoxon rank-sum test nonparametric tests were performed when comparing only two groups and Kruskal-Wallis test followed by Dunn’s post-hoc test when more than two groups were analysed. When comparisons were performed with relative values (percentages), data were previously normalized by using arcsin root transformation. Values of P<0.05 were considered statistically significant. Data are presented as the mean ± standard error of the mean (s.e.m.). In box and whiskers plots, horizontal lines of the box represent Q_3_, median and Q_1_, (+) represents the mean, and whiskers represent maximum and minimum values. Each dot represents an independent culture or animal (*n*), and the *n* is also shown in the graphs. P-values are indicated in the figures. ∗: P<0.05; ∗∗: P<0.01; ∗∗∗: P<0.001; ∗∗∗∗: P<0.0001.

## Supplemental information

Document S1. Figures S1–S9 and Tables S1–S9

Data S1. Results, related to Figures 1, 2, 3, 4, 5, 6, and S1–S9 Source data supporting results.

Data S2. RNAseq analysis, related to Figures 3, 4, 5, S3, S4, and S7 and Tables S1 and S2 Differential expression results obtained with DESeq2. Results have been split into three different tables, corresponding to upregulated, downregulated, and imprinted genes. A thorough description of the results is included in each table.

Data S3. MeDIPseq analysis, related to Figures 4, 5, and S4 and Tables S1 and S2 Differential methylation coverage analysis obtained with the MEDIPS package. Adjacent windows have been merged, and log2 fold changes and adjusted *p*-values have been combined using a harmonic *p*-value approach.
