# Supplementary material for: TET3 protects the Dlk1-Dio3 imprinted locus from DNA hypomethylation during adult NSC reprogramming
Source: iScience. 2025 Nov 12;28(12):113994. doi: 10.1016/j.isci.2025.113994 (PMC12765442; doi:10.1016/j.isci.2025.113994)
Supplement: Document S1. Figures S1–S9 and Tables S1–S9 [file mmc1.pdf]

iScience, Volume *28*

Supplemental information

TET3 protects the *Dlk1-Dio3* imprinted locus from DNA hypomethylation during adult NSC reprogramming

Laura Lázaro-Carot, Esteban Jiménez-Villalba, Jordi Planells, Anna Lozano-Ureña, Jennifer Díaz-Moncho, Raquel Montalbán-Loro, Adela Lleches-Padilla, Martina Kirstein, Mitsuteru Ito, Elizabeth J. Radford, and Sacri R. Ferrón

Supplemental Figures

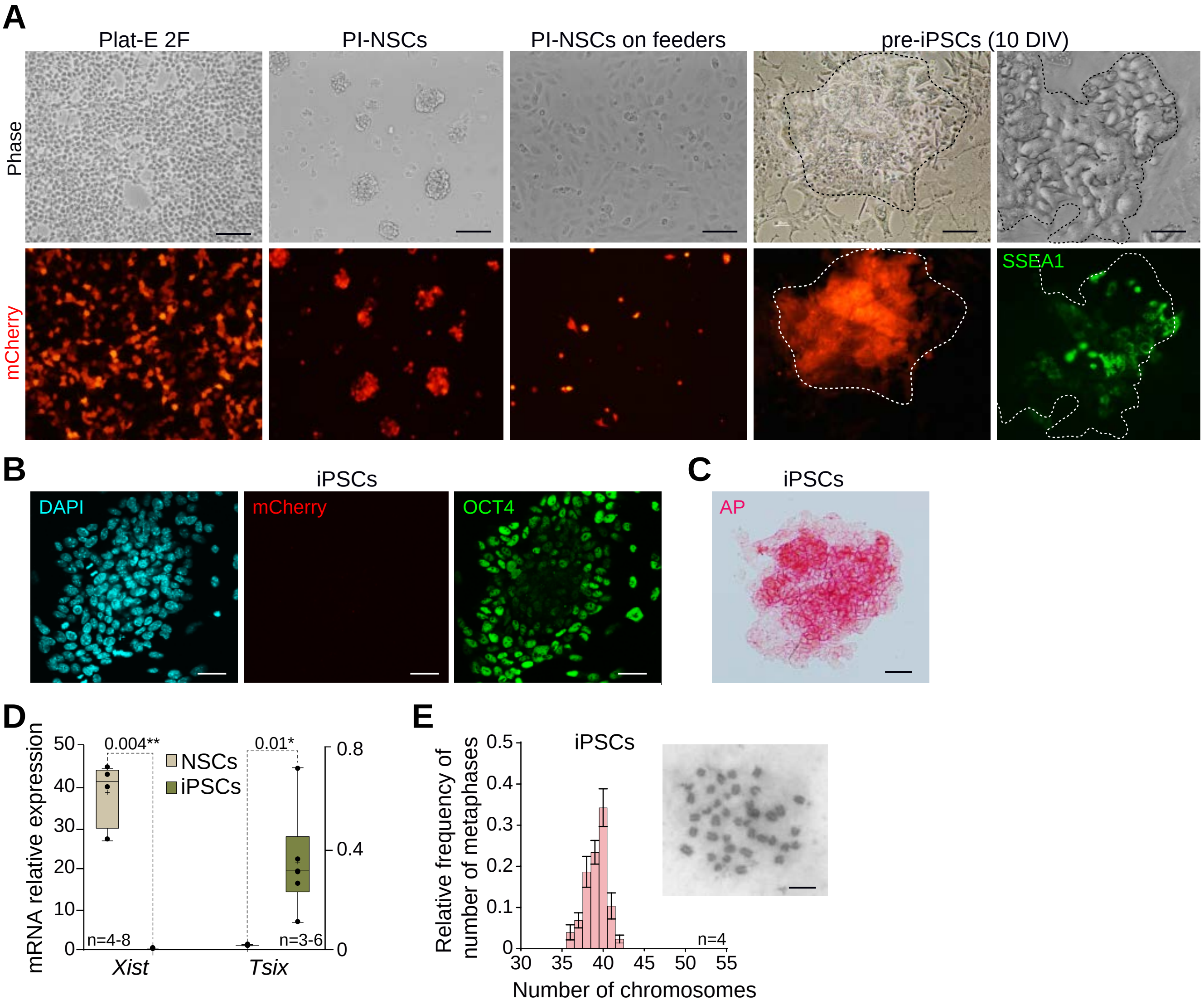

Supplemental Figure 1. Reprogramming of adult NSCs generates iPSCs that express pluripotency genes, related to Fig. 1. **(A)** Phase contrast and fluorescence images showing mCherry expression (red) in Platinum E (Plat-E) retroviral packing cells, post-infected NSCs (PI-NSCs) and pre-iPSCs. Immunocytochemistry (ICC) for SSEA1 (green) in pre-iPSCs cells is shown. **(B)** Fluorescence microscopy images for mCherry (red) and ICC for OCT4 (green) in iPSCs. **(C)** Alkaline phosphatase (AP) staining in iPSCs derived from NSCs. **(D)** qPCR analysis of *Xist* and *Tsix* genes in NSCs and iPSCs. **(E)** Chromosomes counts per metaphase in four selected iPSCs clones. A representative phase-contrast image of Leishman staining is shown. At least 50 metaphases were analyzed per iPSCs line. *Gapdh* was used as a housekeeping gene. DAPI was used for nuclear counterstaining in immunofluorescence images. Scale bars in A: 100 µm (phase contrast) and 10 µm (high magnification images); in B and C: 20 µm; in E: 2 µm. Significance was evaluated using unpaired two-tailed t test. P values and sample sizes are indicated. In bar plots, mean and s.e.m are shown. In box-and-whiskers plots, the mean is marked with a (+) and whiskers represent the minimum and maximum values. Each dot represents an independent culture. *: P<0.05; **: P<0.01; ***: P<0.001; ****: P<0.0001, n.s.: non significant.

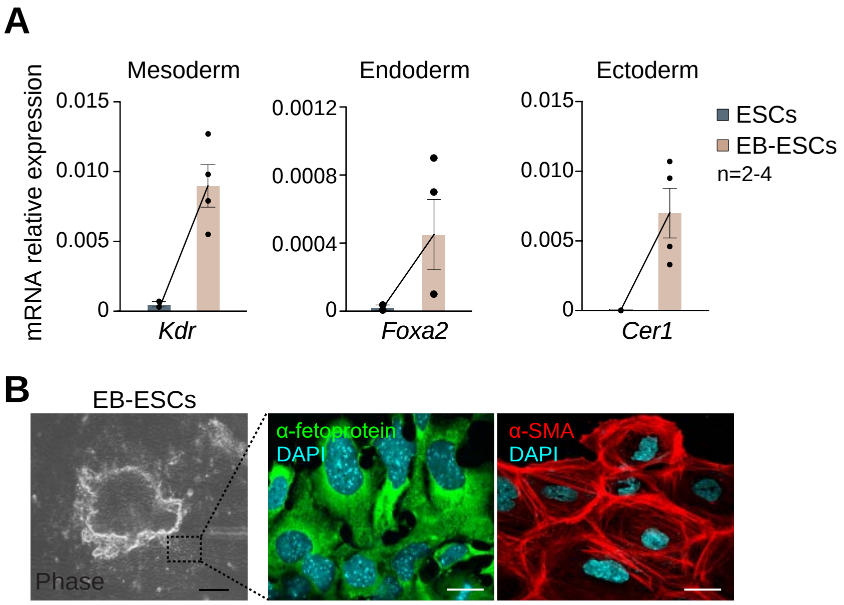

Supplemental Figure 2. Embryoid bodies (EBs) generated from ESCs express markers of the three germ layers, related to Fig. 2. **(A)** qPCR analysis of lineage-specific genes in ESCs and ESC-derived EBs: *Kdr1* (mesoderm), *Foxa2* (endoderm), and *Cer1* (ectoderm). **(B)** Phase contrast image of ESCs-derived EBs (left panel). Immunocytochemistry for α-fetoprotein (green, endoderm) and α-SMA (mesoderm) in EBs. *Gapdh* was used as a housekeeping gene. DAPI was used for nuclear counterstaining. Scale bars in B: 50 μm (phase-contrast image) and 10 μm (fluorescence images). Sample numbers are indicated. In bar plots mean and s.e.m are shown. Each dot represents an independent culture. *: P<0.05; **: P<0.01; ***: P<0.001; ****: P<0.0001, n.s.: non significant.

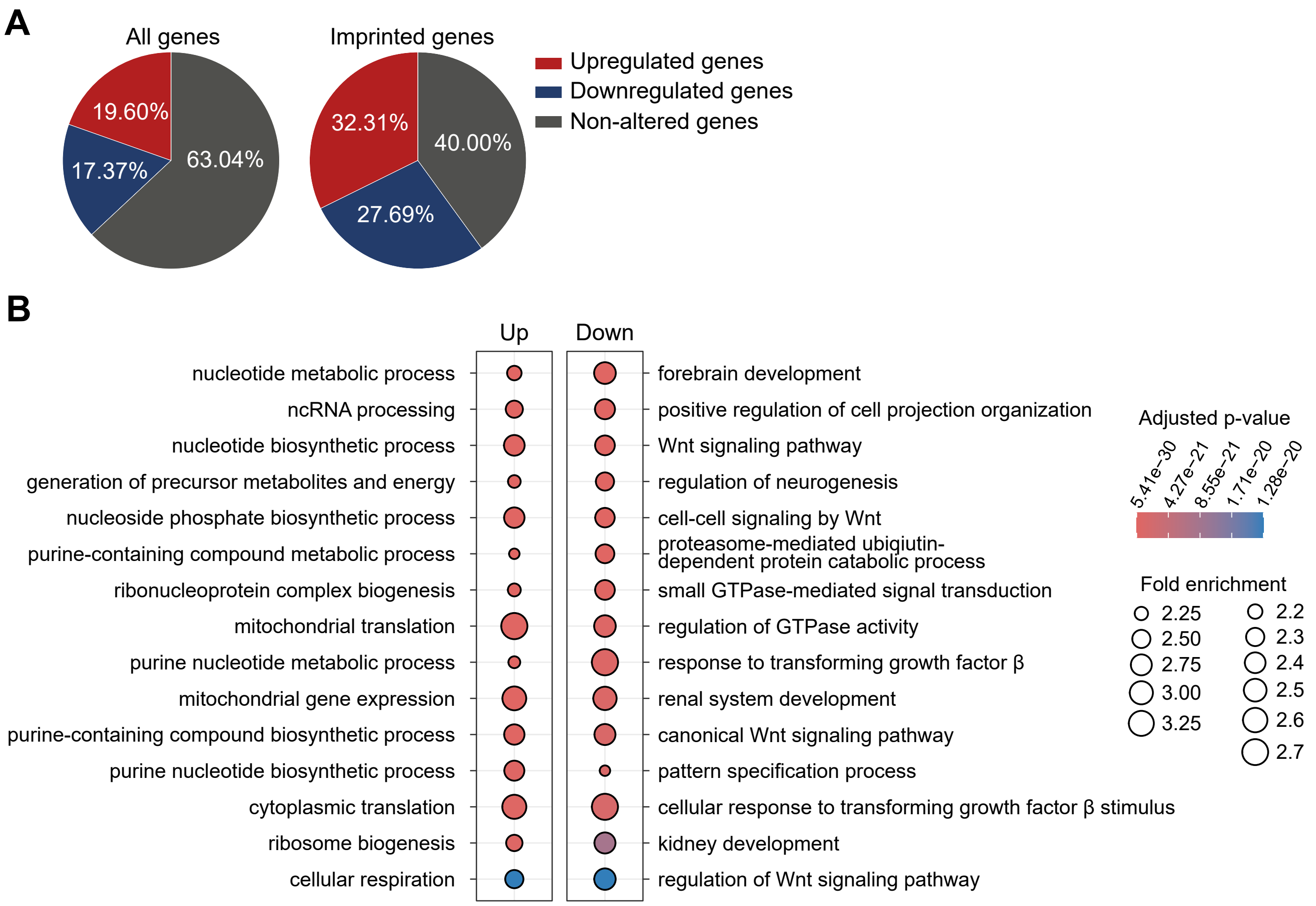

Supplemental Figure 3. Reprogramming of adult NSCs induces global changes in gene expression, related to Fig. 3. **(A)** Pie graphs showing the proportion of all genes and imprinted genes that are upregulated (red), downregulated (blue), or unchanged (gray) in iPSCs relative to adult NSCs. **(B)** Gene ontology (GO) enrichment analysis of biological processes associated with upregulated (Up) and downregulated (Down) genes in iPSCs relative to NSCs.

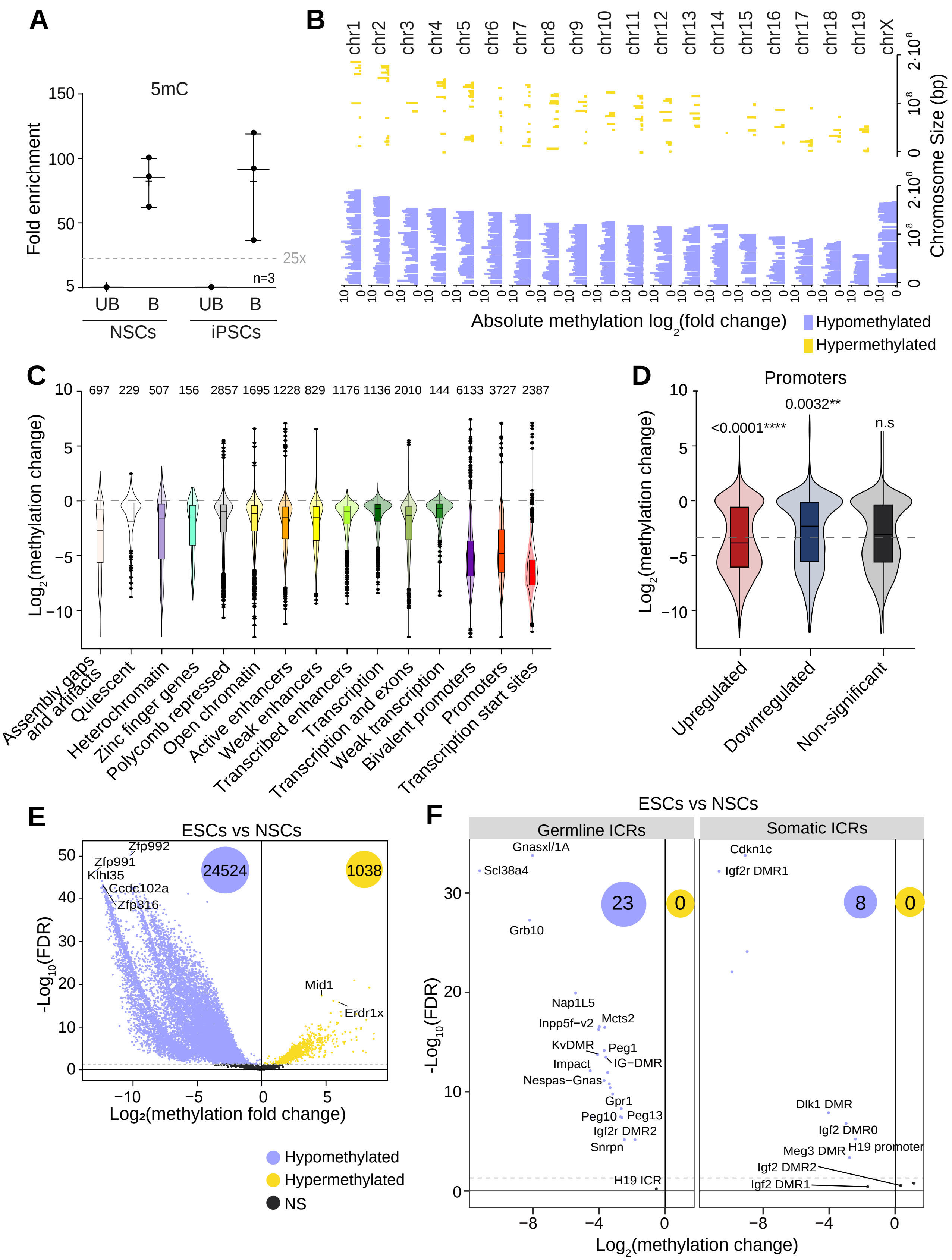

Supplemental Figure 4. Global DNA hypomethylation is observed in iPSCs and ESCs relative to NSCs, related to Fig. 4. **(A)** Fold enrichment control for the MeDIP protocol. Samples were spiked with synthetic *Arabidopsis thaliana* DNA containing 5-methylcytosine (5mC). Only samples with >25x enrichment were selected for sequencing. Bound (B) and unbound (UB) fractions are shown. Mean and s.e.m are shown. **(B)** Genome-wide distribution of DNA methylation changes in iPSCs relative to NSCs. A chromosomal schematic depicts methylation levels. Hypomethylated (purple) and hypermethylated (yellow) regions are shown. **(C)** Violin plots representing $\log_2$(fold change) in methylation levels relative to NSCs across various genomic features and chromatin states. The number of regions overlapping each feature is indicated. **(D)** Violin plots showing $\log_2$(fold change) in promoter methylation of of upregulated, downregulated and unchanged genes in iPSCs relative to NSCs. The dashed gray line indicates the average methylation decrease across all analyzed promoters. **(E)** Volcano plot showing the differential methylation signals between ESCs and NSCs. The number of significantly hypomethylated (purple) and hypermethylated (yellow) regions in ESCs is indicated. Non-significant (NS) changes are shown in black. **(F)** Volcano plot of methylation changes at ICRs between ESCs and NSCs. The number of hypomethylated (purple) and hypermethylated (yellow) DMRs is shown. Significance was evaluated using Wilcoxon rank-sum test. P values and number of samples are indicated. Each dot represents an independent culture. **: P<0.01; ****: P<0.0001, n.s.: non significant.

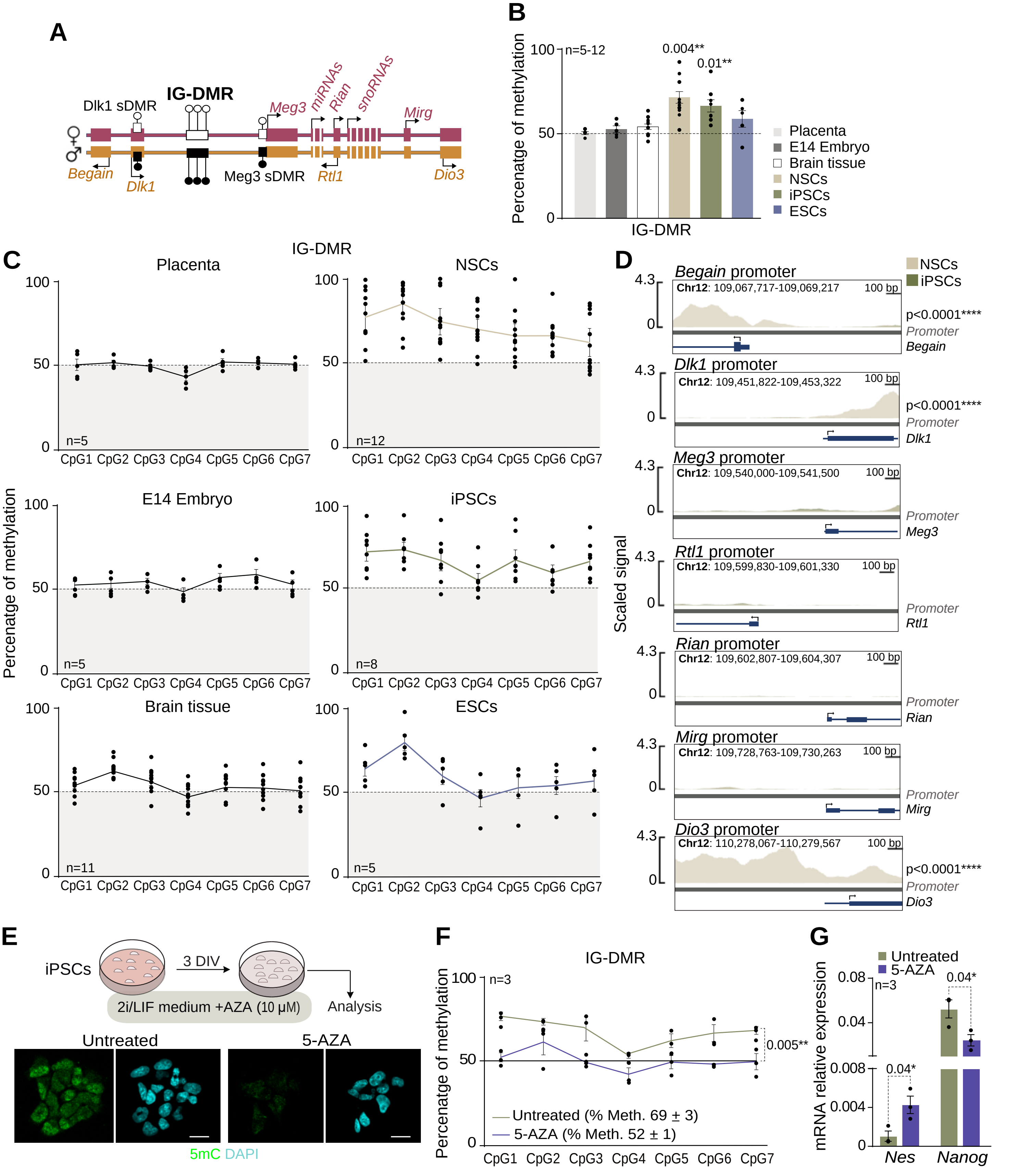

Supplemental Figure 5. Genes located in Dlk1-Dio3 imprinting cluster could be regulated by methylation at their promoters during reprogramming, related to Fig. 5. **(A)** Schematic representation of the Dlk1-Dio3 imprinting cluster. The parent-specific expression of each gene inside the cluster is shown. Methylation status of each DMR is shown by black (methylated) or white (unmethylated) lollipops. **(B)** Average methylation percentage at the IG-DMR in placenta, E14 emrbyos, adult brain tissue, NSCs, iPSCs and ESCs. **(C)** Graph showing methylation percentage at 7 CpGs within the IG-DMR in different tissues and cells. **(D)** MeDIP-seq signal screenshots at the promoter regions of the genes contained in the *Dlk1-Dio3* cluster (*Begain*, *Dlk1*, *Meg3*, *Rtl1*, *Rian*, *Mirg* and *Dio3*) in NSCs and iPSCs. The start and end nucleotide positions based on the Genome Reference Consortium Mouse Build 38 (GRCm38/mm10) are also indicated. **(E)** Schematic of 5-azacytidine (5-AZA) treatment protocol: iPSCs were treated for 3 days with 10μM 5-Aza (upper panel). ICC for 5mC (green) in untreated and 5-AZA-treated iPSCs (lower panel). **(F)** DNA methylation levels at 7 CpGs sites within the IG-DMR, assessed by pyrosequencing of bisulfite-converted DNA in untreated and 5-AZA-treated iPSCs. Average methylation percentage and s.e.m are indicated in brackets. **(G)** qPCR analysis of *Nestin* (Nes) and *Nanog* in untreated and 5-AZA-treated iPSCs. Scale bars in E: 10 μm. Significance was evaluated using unpaired two-tailed t test. P-values and number of samples are indicated. In bar and line plots, mean and s.e.m are shown. Each dot represents an independent culture. *: P<0.05; **: P<0.01; ***: P<0.001; ****: P<0.0001, n.s.: non significant.

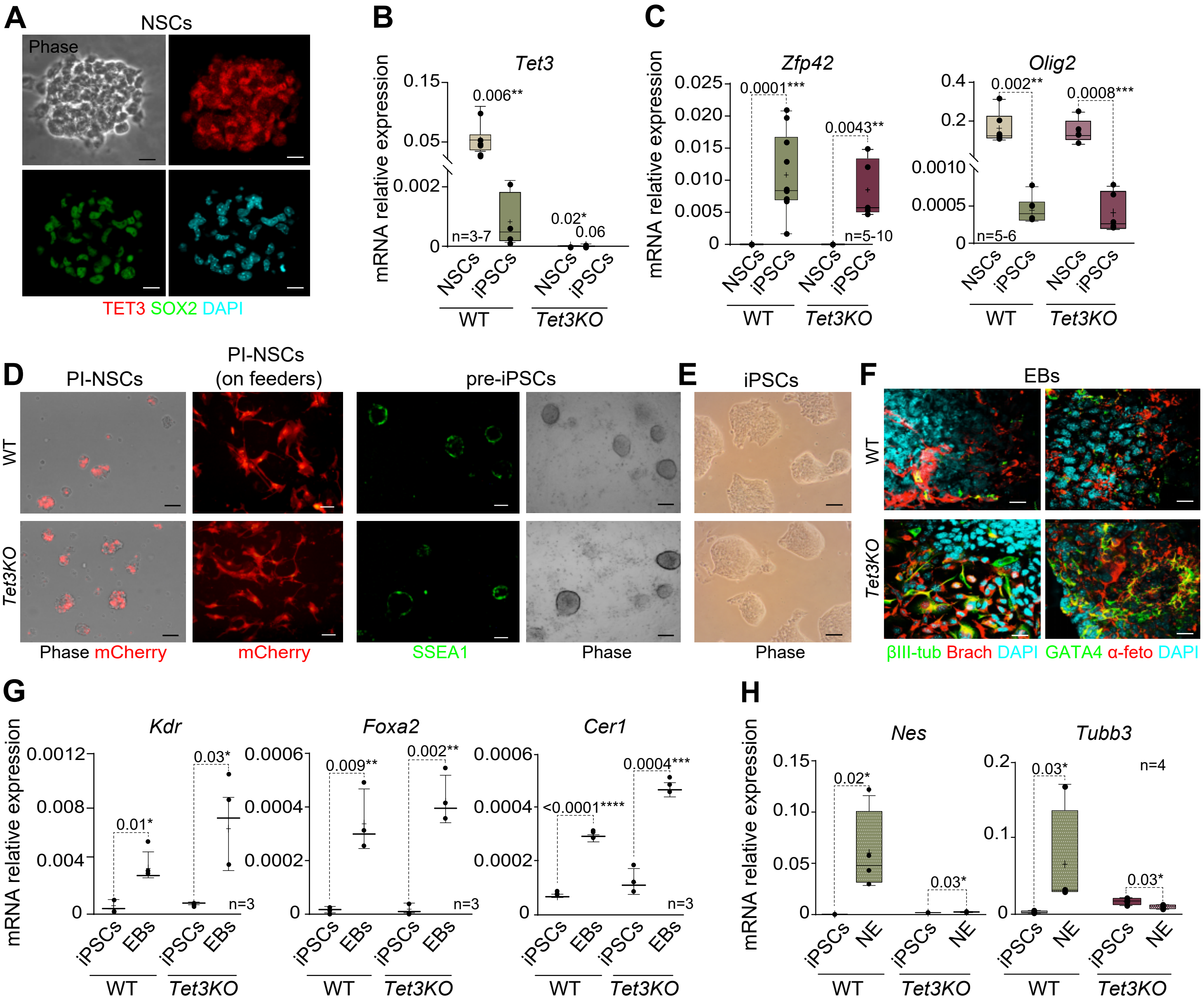

Supplemental Figure 6. iPSCs generated from adult *Tet3*-deficient NSCs differentiate into cells from the three germ layers, related to Fig. 6. **(A)** Phase-contrast and immunocytochemistry (ICC) images of TET3 (red) and SOX2 (green) in wild-type (WT) NSCs. **(B)** qPCR analysis of *Tet3* expression in WT and *Tet3KO* NSCs and iPSCs. **(C)** qPCR of the pluripotency marker *Zfp42* and the neural marker *Olig2* in NSCs and iPSCs of both genotypes. **(D)** Phase-contrast and fluorescence images of mCherry (red) in WT and *Tet3KO* post-infected NSCs (PI-NSCs). Phase-contrast and fluorescence images of SSEA1 (green) in WT and *Tet3KO* pre-iPSCs. **(E)** Phase-contrast images of WT and *Tet3KO* iPSC clones. **(F)** ICC images of βIII-tubulin (green, ectoderm) and Brachyury (red, mesoderm) in EBs from WT and *Tet3KO* iPSCs (left panel), and ICC of GATA4 (green) and α-fetoprotein (red) both endoderm markers (right panel). **(G)** qPCR expression of *Kdr* (mesoderm), *Foxa2* (endoderm), and *Cer1* (ectoderm) in iPSCs and iPSCs-derived EBs of WT and *Tet3KO* cultures. **(H)** qPCR analysis of neural markers *Nes* and *Tubb3* in iPSCs and neurectoderm (NE) derived from WT and *Tet3KO* cultures. *Gapdh* was used as a housekeeping gene for qPCR. DAPI was used to counterstain DNA. Scale bars in A: 10 μm; in D–E: 50 μm; in F: 20 μm. Significance was evaluated using unpaired two-tailed t test. P-values and sample numbers are indicated. In box-and-whiskers plots, the mean is marked with a plus (+) and whiskers represent minimum and maximum values. Each dot represents an independent culture. *: P<0.05; **: P<0.01; ***: P<0.001; ****: P<0.0001.

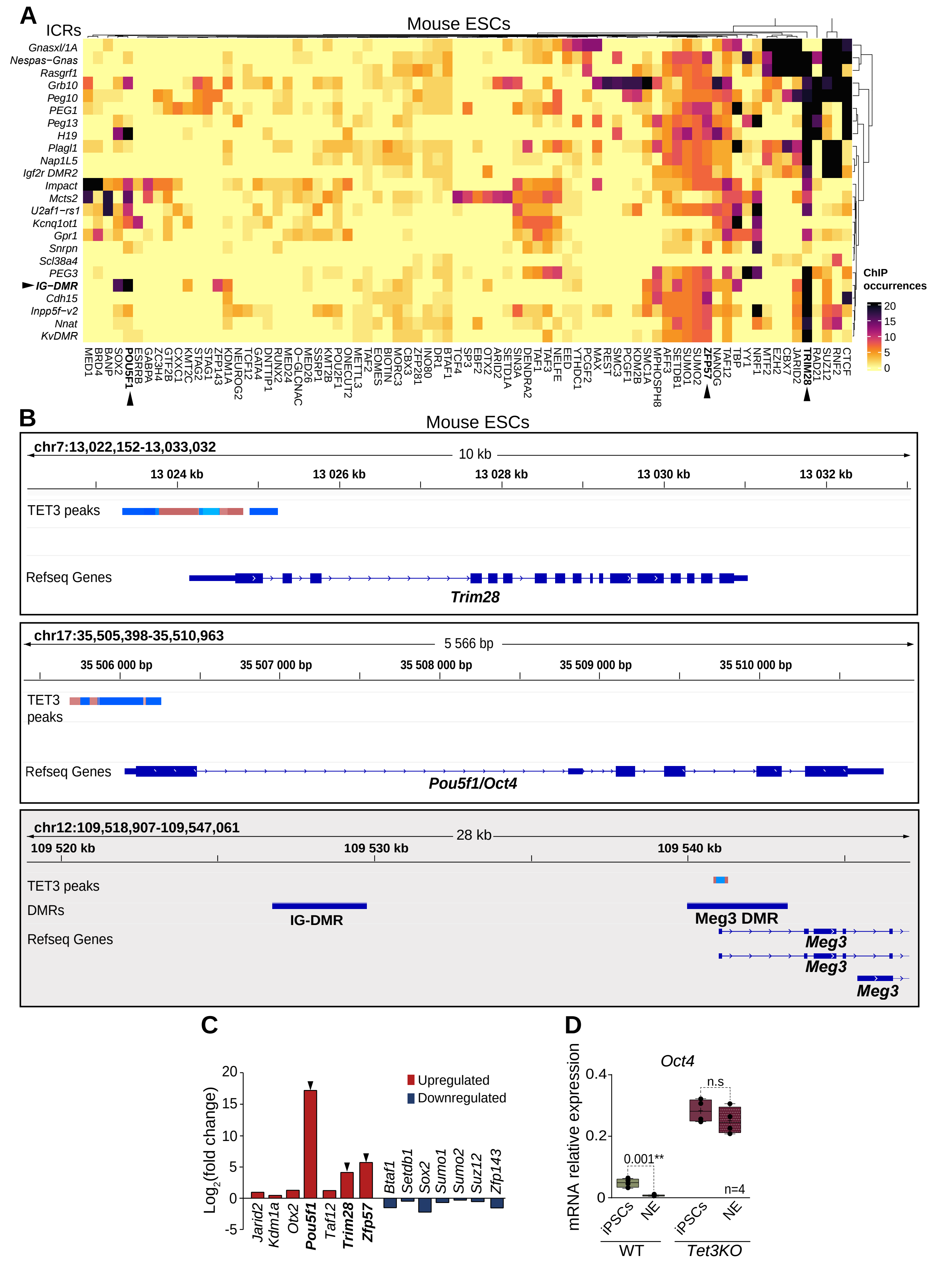

Supplemental Figure 7. TET3 binds *Trim28* and *Oct4* promoters to regulate their expression in mouse ESCs, related to Fig. 6. **(A)** Heatmap showing ChIP-seq peak enrichment for transcription factors and chromatin associated proteins in pluripotent stem cells. Colors represent the frequency of binding (number of peaks) for each protein across different ICRs. **(B)** Genome browser snapshot showing ChIP-seq peaks (in red) indicating TET3 binding at the promoters of *Trim28* and *Oct4* in embryonic stem cells (ESCs). The absence of TET3 peaks near the IG-DMR region (lower panel) is also shown. Data obtained from the ChIP-Atlas database. **(C)** Log_2(fold change) in gene expression from RNA-seq comparing wild type iPSCs to adult NSCs. **(D)** qPCR analysis of *Oct4* expression in iPSCs and neuroectoderm derived from wild-type and *Tet3KO* cultures. Significance was evaluated using unpaired two-tailed t test. P-values and number of samples are indicated. In the box-andwhiskers plot, the mean is indicated by a plus sign (+), and whiskers represent the minimum and maximum values. Each dot represents an independent culture. **: P<0.01; n.s.: non significant.

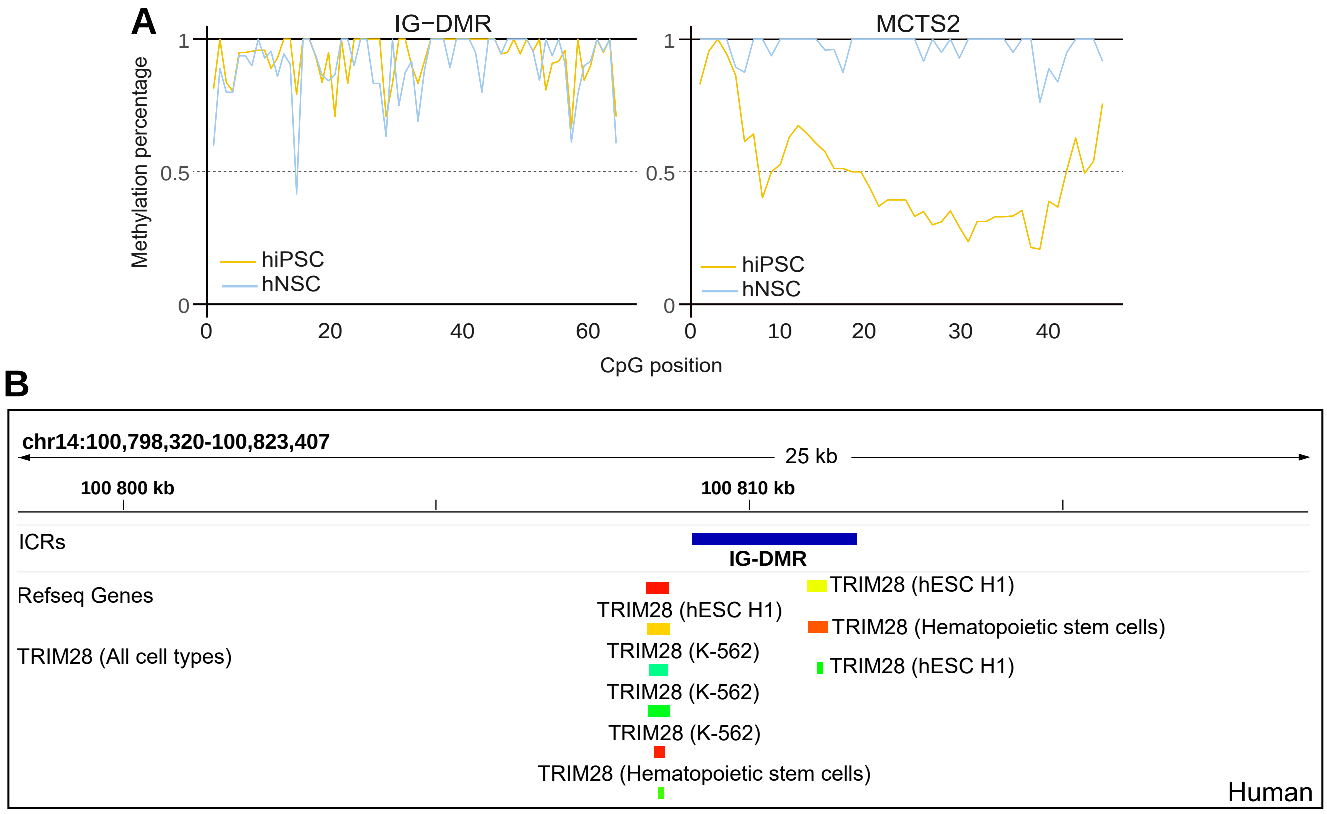

Supplemental Figure 8. IG-DMR hypermethylation is maintained in human pluripotent cells, related to Fig. 6. **(A)** DNA methylation percentage in all CpG nucleotides with bisulfite-sequencing coverage within the IG-DMR in human induced pluripotent stem cells (iPSCs). The same analysis is shown within the human MCTS2 ICR. Data have been accessed through MethBank 4.0. **(B)** Genome browser snapshot showing ChIP-seq peaks (in colours) indicating TRIM28 binding at the IG-DMR region in human ESCs, hematopoietic stem cells, and in the K-562 cell line, derived from lymphoblasts of a chronic myelogenous leukemia patient. TRIM28 profiles have been obtained from ChIP-atlas, using all available cell types, with score = 200.

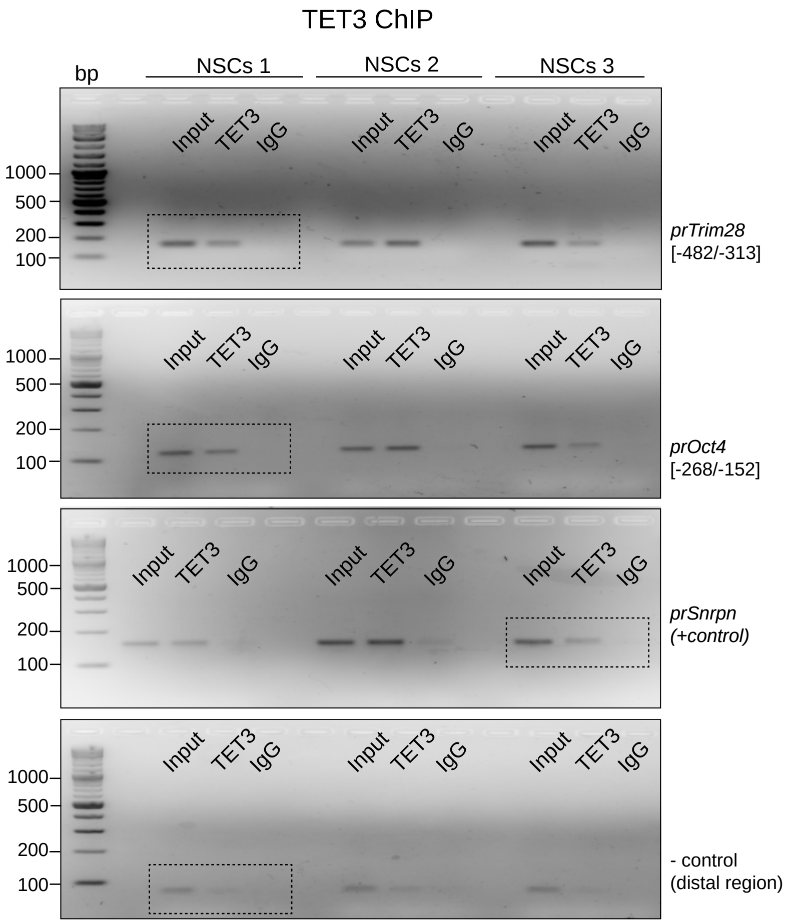

Supplemental Figure 9. Uncropped electrophoresis gel images from qPCR assays following ChIP with TET3 antibody in wild-type adult NSCs, related to Fig. 6. Shown are the full-length gel images corresponding to the data presented in Figure 6G.

Supplemental Tables

Supplemental Table 1. RNAseq and MeDIP-seq results in germline ICRs in iPSCs and adult NSCs.
Germline ICRs methylation changes (MC; edgeR.logFC) and associated p-values (hmp_fdr) from MeDIP-Seq analysis. Hypomethylated and non-altered ICRs are shown in purple and black, respectively. The differential expression (DE; log2 fold changes and p-adjusted values) for genes within each imprinting cluster are also shown. Downregulated genes are indicated in blue and upregulated genes in red. Genes with no significant changes are shown in black. Chromosomic location of each imprinted locus is also listed. NA: not available data.

IMPRINTED LOCUS	ICR	POSITION			iPSC *vs.* NSCs			Genes	iPSCs *vs.* NSCs		
		Chr.	Start	End	LogFC	hmp_fdr	MC		Log2FC	P Adj	DE
**Gpr1/Zdbf2**	Gpr1	chr1	63200357	63200675	-6,56	3,82E-70		*Gpr1*	-5,28	2,83E-80	
								Zdbf2	6,56	5,52E-11	
**Mcts2/H13**	Mcts2	chr2	152686755	152687275	-1,91	0,02		*Mcts2*	1,45	9,22E-08	
								H13	0,15	0,83	
**Nnat**	Nnat	chr2	157560050	157561662	-2,58	7,24E-10		*Nnat*	-2,81	1,28E-12	
								Blcap	-0,82	0,01	
**Gnas**	Gnas1A	chr2	174326930	174329007	-8,22	2,23E-99		*Gnas*	-1,99	7,05E-16	
	Nespas-Gnasxl	chr2	174295707	174300981	-7,19	1,67E-70		*Gnasas1*	1,99	1,27E-05	
**Peg10**	Peg10	chr6	4747209	4748300	-7,05	3,21E-84		*Peg10*	-2,27	0,00	
								Pon1	-3,15	0,17	
								Pon3	-0,80	0,34	
								Ppp1r9a	-3,86	1,39E-07	
								Tfpi2	NA	NA	NA
								Sgce	-1,87	1,08E-06	
								Gng11	-5,96	1,69E-05	
								Asb4	-1,65	0,05	
								Dlx5	3,40	0,13	
								Calcr	4,52	0,02	
								Pon2	-6,24	2,23E-40	
**Mest**	Peg1	chr6	30736488	30739335	-6,21	1,71E-84		*Mest*	-3,87	1,25E-10	
								Cpa4	-1,24	0,22	
								Copg2	0,82	0,02	
								Klf14	5,09	0,04	
**Nap1L5**	Nap1L5	chr6	58906696	58907062	-7,41	1,11E-77		*Nap1L*	-1,65	0,30	
								Aqp1	-2,45	0,10	
**Peg3**	Peg3	chr7	6729433	6730510	-6,86	2,10E-70		*Zim1*	-1,99	0,13	
								Zim2	NA	NA	NA
								Zim3	NA	NA	NA
								Peg3	-0,67	0,14	
								Usp29	1,10	0,19	
								Zfp264	9,99	1,24E-11	
								Peg3os1	NA	NA	NA
**Snrpn**	Snurf-Snrpn	chr7	60004992	60005415	-6,19	9,76E-33		*Peg12*	-5,70	4,76E-31	
								Mkrn3	-1,46	1,69E-06	
								Magel2	9,94	2,40E-12	
								Ndn	8,07	1,98E-12	
								Snrpn	12,04	3,37E-20	
								Snurf	3,82	2,15E-89	
								Ube3a	-3,21	1,74E-37	
								Atp10a	-1,26	0,0002	
**Inpp5f**	Inpp5f-v2	chr7	128687968	128688584	-6,81	9,94E-67		*Inpp5f*	-3,01	1,20E-05	
**H19/Igf2**	H19	chr7	142580263	142582695	-1,97	0,001		*H19*	8,10	8,36E-16	
								Igf2	5,82	1,16E-07	
								Igf2os1	0,17	0,95	
								Ins2	NA	NA	NA

Supplemental Table 1. Continued.

IMPRINTED LOCUS	ICR	POSITION			iPSCs vs. NSCs			Genes	iPSC vs. NSCs		
		Chr.	Start	End	LogFC	hmp_fdr	MC		Log2FC	P Adj	DE
								Th	11,29	4,02E-17	
								Tssc4	1,13	1,34E-06	
								Kcnq1	NA	NA	NA
								Kcnq1ot1	-3,42	2,33E-19	
								Cdkn1c	1,04	0,33	
								Slc22a18	7,48	5,80E-38	
**Kcnq1 Kcnq1ot1 Lit1**	KvDMR	chr7	143295309	143295849	-8,98	3,20E-80		*Phlda2*	11,63	2,37E-34	
								Nap1l4	-1,37	0,003	
								Osbpl5	-3,40	3,20E-16	
	Ex1_Kcnq1ot1	chr7	143296095	143297095	-8,98	3,20E-80		*Trpm5*	4,00	0,05	
								Tnfrsf22	2,77	6,13E-17	
								Tnfrsf23	1,67	0,01	
								Tnfrsf26	-0,98	0,36	
								Ascl2	4,28	1,26E-14	
								Tspan32	6,07	2,12E-61	
								Cd81	-2,78	5,40E-32	
**Cdh15**	Cdh15	chr8	122864539	122865917	-1,21	1,04E-07		*Cdh15*	6,64	3,28E-09	
**Rasgrf1**	Rasgrf1	chr9	89876812	89885505	-8,20	3,26E-59		*Rasgrf1*	7,52	0,0001	
**Plagl1**	Plagl1	chr10	13090437	13091326	-8,75	2,45E-99		*Plagl1*	2,10	1,03E-11	
								Grb10	8,51	1,62E-79	
**Grb10**	Grb10	chr11	12025515	12026351	-8,19	3,32E-81		*Cobl*	10,08	3,06E-79	
								Ddc	7,92	1,23E-49	
**Commd1**	U2af1-rs1	chr11	22971895	22973136	-11,32	2,19E-57		*Commd1*	0,97	0,02	
								Zrsr1	0,09	0,68	
								Begain	6,38	1,48E-06	
								Dlk1	10,69	6,79E-15	
								Meg3	11,72	1,58R-07	
**Dlk1/Meg3**	IG-DMR	chr12	109526740	109529760	0,29	0,24		*Rtl1*	6,73	0,05	
								Dio3	13,39	1,72E-24	
								Mirg	11,02	0,003	
								Rian	8,50	2,11E-06	
								Kcnk9	10,04	3,15E-06	
								Trappc9	0,87	1,81E-07	
**Peg13**	Peg13	chr15	72808840	72810685	-5,72	8,02E-45		*Peg13*	-0,02	0,99	
								Ago2	-0,91	0,20	
								Chrac1	0,36	0,24	
**Slc38a4**	Scl38a4	chr15	97054197	97055313	-7,63	4,70E-90		*Slc38a4*	14,39	7,87E-35	
								Slc22a1	NA	NA	NA
								Slc22a2	0,22	0,95	
**Igf2r**	Igf2r DMR2	chr17	12741297	12742707	-2,04	0,0002		*Slc22a3*	2,07	0,49	
								Igf2r	-1,79	4,98E-07	
								Airn	-5,37	3,11E-08	
								Mas1	4,70	0,002	
**Impact**	Impact	chr18	12971749	12974325	-6,82	6,57E-78		*Impact*	-1,26	3,08E-07	

Supplemental Table 2. RNAseq and MeDIP-seq results in somatic ICRs in iPSCs and adult NSCs. Somatic ICR methylation changes (MC; edgeR.logFC) and associated p-values (hmp_fdr) from MeDIP-Seq analysis. Hypomethylated and hypermethylated ICRs are shown in purple and yellow, respectively. Non-altered ICRs are indicated in black. The related genes and the chromosomic location of each imprinted locus is also listed.

IMPRINTED LOCUS	ICR	POSITION			iPSC *vs.* NSCs			Related Genes
		Chr.	Start	End	LogFC	hmp_fdr	MC	
**Zdbf2**	Zdbf2 DMR1 amplicon 3	Chr1	63257407	63257858	0,34	0,87		*Zdbf2*
	Zdfb2 DMR1 amplicon 7	Chr1	63257407	63257858	0,34	0,87		
	Zdbf2 DMR2 amplicon 11	Chr1	63257407	63257858	0,34	0,87		
**Gnas**	Nesp	Chr2	174284269	174286690	-9,07	1,03E-100		*Gnas*
**Snrpn**	Ndn	Chr7	62348252	62348745	-10,27	2,92E-54		*Ndn*
**H19/Igf2**	H19 promoter	Chr7	142578136	142578879	-2,82	9,60E-13		*H19* *Igf2*
	Igf2 DMR0	Chr7	142669087	142578879	-0,18	0,70		
	Igf2 DMR1	Chr7	142665179	142665719	-1,97	0,05		
	Igf2 DMR2	Chr7	142653809	142654708	2,86	9,74E-20		
**Kcnq1 Kcnq1ot1 Lit1**	Cdkn1c	Chr7	143459053	143461485	-7,63	2,14E-89		*Cdkn1c*
**Dlk1/Meg3**	Dlk1 DMR	Chr12	109459858	109460079	-1,56	3,81E-11		*Dlk1*
	Meg3 DMR	Chr12	109539990	109543190	1,14	0,03		*Meg3*
**Igf2r**	Igf2r DMR1	Chr17	12769042	12770027	-6,82	1,13E-109		*Igf2r*

Supplemental Table 3. List of primary antibodies used.
ICC: immunocytochemistry; ChIP: chromatin immunoprecipitation.

Primary antibody	Source	Host	Dilution	Cat. no.	Application
5mC	Diagenode	Mouse	1:1000	C15200006	ICC
α-fetoprotein	R&D	Rabbit	1:100	mab1368	ICC
βIII-tubulin	Covance	Mouse	1:300	PRB-435P	ICC
Brachyury	Santa Cruz	Goat	1:500	sc-17743	ICC
GATA-4	Santa Cruz	Goat	1:1000	sc-1237	ICC
IgG	Santa Cruz	Rabbit	1:20	sc-2027	ChIP
NANOG	Reprocell	Rabbit	1:100	RCAB002PF	ICC
Nestin	Hybridoma Bank	Mouse	1:4	rat-401	ICC
OCT-4	Santa Cruz	Rabbit	1:200	sc-5279	ICC
OLIG2	Millipore	Rabbit	1:500	AB9610	ICC
α-Smooth Muscle Actin (α-SMA)	Abcam	Mouse	1:100	ab18147	ICC
SOX2	R&D Systems	Goat	1:200	AF2018	ICC
SSEA1	Stemgent	Mouse	1:100	09-0067	ICC
TET3	Millipore	Rabbit	1:100/1:50	ABE290	ICC/ChIP

Supplemental Table 4. List of secondary antibodies used for immunocytochemistry.

Secondary antibody	Source	Dilution	Cat. no.
Alexa Fluor® 488 Donkey Anti-Mouse	Molecular Probes	1:1000	A-21202
Alexa Fluor® 488 Donkey Anti-Rabbit	Jackson ImmunoResearch	1:1000	711-547-003
Alexa Fluor® 647 Donkey Anti-Rabbit	Jackson ImmunoResearch	1:1000	711-607-003
Cy3-Donkey Anti-Rabbit	Jackson ImmunoResearch	1:2000	711-165-152
Cy3-Donkey Anti-goat	Jackson ImmunoResearch	1:2000	705-166-147
Cy3-Donkey Anti-Mouse	Jackson ImmunoResearch	1:2000	715-165-151

Supplemental Table 5. List of TaqMan probes used. From Applied Biosystems. *TaqMan probes designed by us.

Gene	Taqman code	Gene	Taqman code
Afp	Mm00431715_m1	*Oct4*	Mm00658129_gH
Cer1	Mm00515474_m1	*Olig2*	Mm01210556_m1
c-Myc	Mm00487803_m1	*Pax6*	Mm00443081_m1
Cobl	Mm01187905_m1	*Peg10*	Mm01167724_m1
Dio3	Mm00548953_s1	*Peg12*	Mm00844053_s1
Dlk1	Mm00494477_m1	*Phlda2*	Mm00493899_g1
Foxa2	Mm01976556_s1	*Plagl1*	Mm00494251_m1
Gnas	Mm01242435_m1	*Ppp1r9a*	Mm00725102_m1
Gapdh	Mm99999915_g1	*RETRO Klf4**	FAM-CCCCTTCACCATGGCTG-MGB
H19	Mm01156721_g1	*RETRO Oct4**	FAM-CACCTTCCCCATGGCTG-MGB
Igf2	Mm00439564_m1	*Slc38a4*	Mm00459056_m1
Kdr	Mm01222421_m1	*Sox2*	Mm03053810_s1
Klf4	Mm00516104_m1	*Th*	Mm00447557_m1
Meg3	Mm03456293_m1	*Trim28*	Mm00495594_m1
Meox1	Mm00440285_m1	*Zdbf2*	Mm01254509_m1
Nanog	Mm02384862_g1	*Zfp42*	Mm01194089
Nes	Mm00450205_m1	*Zfp57*	Mm00456405_m1_
Ndn	Mm02524479_s1	*Zic1*	Mm00656094_m1

Supplemental Table 6. List of Syber Green primers used.

Gene	Forward (FW)	Reverse (RW)
Tsix	TGTCAGGTTTCGGGGACACT	CTCTCCAGCCCAGGAACTGA
Xist	CTCATAGTAGTGGCCGACTA	TAAGCCCGTTAAGTAGTCCTT
Tet3	AGTGGGTGATCCGAAGACAC	TGTTAGGGTCTTTGCCTTGG
prTrim28 (ChIP)	AGTCAAGATTCCTCCCCAGGTCTT	GCCCCTTCTGTGTTAGTCCTCTGCTA
prOct4 (ChIP)	GCAGTGCCAACAGGCTTTGT	CCAGGAGGCCTTCATTTTCAACCT
prSnrpn (ChIP)	ACTCCTTGGGTGTGTTAGTG	GACTTCCAGGAGTCCAGAGG
Distal region (ChIP)	GACAGCAGCCGAACTTCGTT	TGGTGCCCGAGGAAGATG

Supplemental Table 7. Primers used to evaluate MeDIP-seq enrichment and specificity.

Region	Forward (FW)	Reverse (RW)
Methylated region	CATGGCCCACAAAGTAATAAAA	AACGACTTACAACGAGCTCAAA
Unmethylated region	GGCTAGAACTGACCAGACAGAC	ATCTGTAGCCAATCCTAGAGCA

Supplemental Table 8. List of pyrosequencing primers.

DMR	Forward (FW)	Reverse (RW)	Sequencing
IG-DMR	GTGGTTTGTTATGGGTAAGTTT	CCCTTCCCTCACTCCAAAAATTAA	GGTAAGTTTTATGGTTTATTGTATA
Igf2r DMR2	GGGTGAAGATTTTTGGGTTATAAG	CCCCCCCCAATACAACAA	TTTATTGTTTATTAGTGTTTTGAAT
KvDMR	AGAAGGGTGTTGAAGAAAAATT	ATCCTAAACCTAAACCTCCATAA	GTTGAGAAGTTAAGTGGA
Mcts2 DMR	TGAAGAAGAATTAGTGGGGTAA	ACAATTAAACACACTTTCCTTCTC	GGTGTTATTTTTTTTGTAGA
Peg10 DMR	AATTTTGTTAAGTTTTTAGTGGTTAGAT	CACTTAAAAATACAAAACCAATCACTT	CACAATTCCATCAATAACT
Snurf-Snrpn DMR	TTGGTAGTTGTTTTTTGGTAGGAT	TCCACAAACCCAACTAACCTTC	GTGTAGTTATTGTTTGGGA
Zrsr1 DMR	ATGGTTAGGTTGAGAGTTTTGGAAGTTT	TCCCTCAACAACCACTCTTCATA	TTTTGGAAGTTTTATTAGAGG

Supplemental Table 9. List of software and tools used in this study. Package name and version, and the repository for the different tools are indicated.

Package/Tool	Package Version	Repository	Reference
ChIPSeeker	1.38.0	https://bioconductor.org/packages/ChIPseeker	79
clusterProfiler	4.10.1	https://bioconductor.org/packages/clusterProfiler	70
ComplexHeatmap	2.18.0	https://bioconductor.org/packages/ComplexHeatmap	72
Deeptools	3.5.4	https://github.com/deeptools/deepTools	75
DESeq2	1.42.1	https://bioconductor.org/packages/DESeq2	69
dplyr	1.1.4	https://cloud.r-project.org/web/packages/dplyr/index.html	71
edgeR	4.0.16	https://bioconductor.org/packages/edgeR	77
ggplot2	3.5.1	https://cloud.r-project.org/web/packages/ggplot2/index.html	71
MEDIPS	1.54.0	https://bioconductor.org/packages/MEDIPS	78
MEDUSA	1.0.0	https://www.ucl.ac.uk/cancer/research/department-cancer-biology/medical-genomics-past-projects/medusa	74
plyranges	1.22	https://www.bioconductor.org/packages/plyranges	80
Salmon	1.10.1	https://github.com/COMBINE-lab/salmon	66
SparK	2.6.2	https://github.com/harbourlab/SparK	81
tibble	3.2.1	https://CRAN.R-project.org/package=tibble	71
tidyr	1.3.1	https://CRAN.R-project.org/package=tidyr	71
tximeta	1.20.3	https://CRAN.R-project.org/package=dplyr	68
